# Making watercress (*Nasturtium officinale*) cropping sustainable: genomic insights into enhanced phosphorus use efficiency in an aquatic crop

**DOI:** 10.3389/fpls.2023.1279823

**Published:** 2023-11-07

**Authors:** Lauren E. Hibbert, Yufei Qian, Hazel K. Smith, Suzanne Milner, Ella Katz, Daniel J. Kliebenstein, Gail Taylor

**Affiliations:** ^1^ Department of Plant Sciences, University of California Davis, Davis, CA, United States; ^2^ School of Biological Sciences, University of Southampton, Hampshire, United Kingdom; ^3^ Vitacress Salads Ltd, Andover, United Kingdom

**Keywords:** abiotic stress, Brassica, fertilizer, nutrition, Nasturtium officinale, phosphorus, transcriptome

## Abstract

Watercress (*Nasturtium officinale*) is a nutrient-dense salad crop with high antioxidant capacity and glucosinolate concentration and with the potential to contribute to nutrient security as a locally grown outdoor aquatic crop in northern temperate climates. However, phosphate-based fertilizers used to support plant growth contribute to the eutrophication of aquatic habitats, often pristine chalk streams, downstream of farms, increasing pressure to minimize fertilizer use and develop a more phosphorus-use efficient (PUE) crop. Here, we grew genetically distinct watercress lines selected from a bi-parental mapping population on a commercial watercress farm either without additional phosphorus (P−) or under a commercial phosphate-based fertilizer regime (P+), to decipher effects on morphology, nutritional profile, and the transcriptome. Watercress plants sustained shoot yield in P− conditions, through enhanced root biomass, but with shorter stems and smaller leaves. Glucosinolate concentration was not affected by P− conditions, but both antioxidant capacity and the concentration of sugars and starch in shoot tissue were enhanced. We identified two watercress breeding lines, with contrasting strategies for enhanced PUE: line 60, with highly plastic root systems and increased root growth in P−, and line 102, maintaining high yield irrespective of P supply, but less plastic. RNA-seq analysis revealed a suite of genes involved in cell membrane remodeling, root development, suberization, and phosphate transport as potential future breeding targets for enhanced PUE. We identified watercress gene targets for enhanced PUE for future biotechnological and breeding approaches enabling less fertilizer inputs and reduced environmental damage from watercress cultivation.

## 1 Introduction

Watercress (*Nasturtium officinale* R. Br.) is a perennial leafy green crop adapted to growth in aquatic environments. Found in nature in free-flowing alkaline streams and as a crop, it is best grown in hydroponic systems, including open water ponds, hydroponic greenhouses, and vertical farms (Cox, 2009; Schuchardt et al., 2019; Qian et al., 2022). Watercress is a member of the Brassicaceae family, alongside other important food crops such as broccoli (*Brassica oleracea* var. *italica*) and oilseed rape (*B. napus*) (Kiefer et al., 2019). A distinctive characteristic of watercress is its peppery flavor, derived from the hydrolysis of gluconasturtiin, the primary glucosinolate (GSL) in watercress, to phenethyl isothiocyanate (PEITC) (Boyd et al., 2006; Payne, 2011; Jeon et al., 2017). Although the primary function of glucosinolates is for defense against herbivory, it is the isothiocyanates that are responsible for the anti-cancer, antibiotic, and cardioprotective properties of watercress (Newman et al., 1992; Cheung and Kong, 2009; Panahi Kokhdan et al., 2021). Watercress also has high antioxidant (AO) capacity and qualifies as the most nutrient-dense fruit/vegetable, based on the content of 17 nutrients of public health importance (Di Noia, 2014).

Watercress is grown worldwide, including the UK, USA, Spain, Portugal, New Zealand, and China (Li et al., 2007b; Searle, 2019; USDA, 2019). In the UK, 58 hectares of watercress were grown in 2018, representing a total value of £15 million (DEFRA, 2020). It is also a high-value horticultural crop, with a UK market value of £8.90 per kg compared to £4.97 per kg for mixed baby leaf salad bags (DEFRA, 2019). However, there is concern that watercress production is causing environmental pollution, through the direct addition of phosphate-based fertilizers into aquatic systems, including chalk streams in the UK, which are of high conservation value (Cox, 2009; Hibbert and Taylor, 2022). Chalk streams are internationally rare and highly biodiverse environments often referred to as “England’s rainforests”, providing a habitat for species such as the winterbourne stonefly (*Nemoura lacustris*) and brown trout (*Salmo trutta*), which depend on its clean low nutrient waters—but they are under threat (White, 2020; CaBA CSRG, 2021; Environment Agency and Natural England, 2021). In these and other aquatic systems, phosphate pollution results in eutrophication of water systems, where excessive flora growth limits light penetration, leading to death of organisms below, deoxygenation of the habitat via microbial decomposition, and ultimately the disruption of community dynamics (Schindler et al., 2008). Naturally, phosphate concentrations in chalk streams are approximately 0.02 mg L^−1^; however, inputs of phosphorus (P) rapidly increase these concentrations above P targets downstream of watercress farms (Casey and Smith, 1994). Additional P inputs into freshwater systems is predominantly through release from sewage treatment works (STWs), leaking septic tanks, and from excess fertilizer application (Withers et al., 2013; Richards et al., 2016; CaBA CSRG, 2021). However, watercress farms were also shown to contribute 5.4% of the P load in chalk streams and values of up to 62% were reported for some streams, suggesting significant room for improvement (Cox, 2009). Approximately 90% of watercress farms in the UK are on, or upstream of, a Site of Special Scientific Interest (SSSI), increasing the pressure to minimize Prelease. Of the 249 chalk streams in the UK, 39% fail the standards for good ecological P status set out in the Water Framework Directive (European Commission, 2019). Jarvie et al. (2018) also surveyed the nutrient status of British headwater streams and declared that reducing P concentrations in lowland-high-alkalinity rivers (chalk streams) is one of the most important areas to target for improved UK water quality.

P is vital for plant growth, and is used to power cells through the release of phosphate from ATP, for the structure of protein and carbohydrate polymers, cell membranes, and the formation of the phosphodiester bonds that link nucleotides (Westheimer, 1987; Schachtman et al., 1998). Nevertheless, the environmental damage associated with phosphate fertilizer application and the finite nature of P reserves is driving the development of crops with improved P-use efficiency (PUE). For watercress, with commercial production linked to chalk streams, improved PUE is a key target for crop improvement (Hibbert and Taylor, 2022). Our recent review identified the key traits to breed for a PUE ideotype in watercress as (i) increased root surface area through prolific root branching, adventitious root (free floating roots deriving from the stem) formation, and root hair growth, and (ii) increased root aerenchyma formation. Functional genomic traits for improved PUE are (iii) efficacious premobilization and scavenging strategies and (iv) the use of alternative metabolic pathways (Hibbert and Taylor, 2022). Identifying gene-based targets central to the PUE response in watercress is vital to increase the speed of breeding for PUE. Key genomic targets in other species (predominantly based on studies in the soil-grown model plant, *Arabidopsis thaliana*) have previously been identified as *PHT* phosphate transporter genes, global transcriptional regulators such as those of the *SPX* family, and genes involved in galactolipid and sulfolipid biosynthesis such as *MGD2/3*, *PECP1, PSR2, PLDζ1/2*, and *SQD2* (Hibbert and Taylor, 2022). Although matches for these genes have been found in watercress transcriptome data, the functional significance of these gene targets, particularly in P-deficient growing environments, have not yet been investigated. Other breeding targets for consumers include further improved nutritional quality (AO capacity and GSL concentration) and enhanced sweetness while maintaining yield, and it is therefore important to understand the trade-offs between fertilizer management and nutritional profile, yield, and crop flavor.

There is limited understanding of the effects of nutrient availability on watercress. One study utilized an experimental stream to assess differences in watercress growth rate under varying N:P application, with a focus on N accumulation (Fernandez-Going et al., 2013). As expected, growth rates increased with increasing nutrient availability. Previously, microarray and RNA-sequencing (RNA-seq) approaches have been used to explore differences in AO capacity and GSL concentration, and to study molecular mechanisms underlying contrasting growth responses to submergence in watercress (Payne, 2011; Voutsina et al., 2016; Jeon et al., 2017; Müller et al., 2021). Authors used orthology to *A. thaliana* to identify key candidate genes involved in GSL/AO biosynthesis pathways and hormone signaling mediating growth responses.

There is an urgent need, therefore, to identify gene targets to enable future breeding for PUE in this nutrient dense leafy green crop. There is currently no relevant breeding for PUE and the literature surrounding the effects of fertilizer on watercress growth is limited and outdated (Austin, 1966; Howard-Williams et al., 1982; Bennett, 1986; Fernandez-Going et al., 2013). This study aims to investigate the genomic basis of PUE in watercress, through a study of the effects of contrasting applications of phosphate-based fertilizer on the growth, biochemistry, and gene expression of selected watercress lines from a bi-parental mapping population using RNA-seq approaches.

## 2 Materials and methods

### 2.1 Plant material

An F_2_ watercress mapping population was previously developed by crossing two lines contrasting for size and nutritional content: WX033 and WX038 (also referred to as Parent A and Parent B, respectively, in Voutsina, 2017). WX033 is the commercialized dwarf leafy “Boldrewood” cultivar with high AO and GSL concentration. WX038 is an accession with a longer stem and lower AO and GSL than WX033 in both field and controlled conditions (Payne, 2011). F_2_ offspring were self-fertilized to obtain the F_3_ (F_2:3_) generation, then multiple plants were grown and seed harvested in bulk (F_2:4_) (Qian, 2021). This enabled greater seed production for use in this field trial, with a similar homozygosity. Nine watercress lines (referred to as 120, 102, 39, 82, 60, 225, 5, 16, and 173) from this watercress mapping population, the two parent lines (WX033 and WX038), and two commercial control lines (WX001 and WXVITA) were selected for this field study. These lines were selected based on high GSL concentration, AO capacity, vigor, and desirable morphological traits for commercial cultivation observed in previous trials (Qian, 2021; Qian et al., 2023).

### 2.2 Experimental design

**Figure 1:**
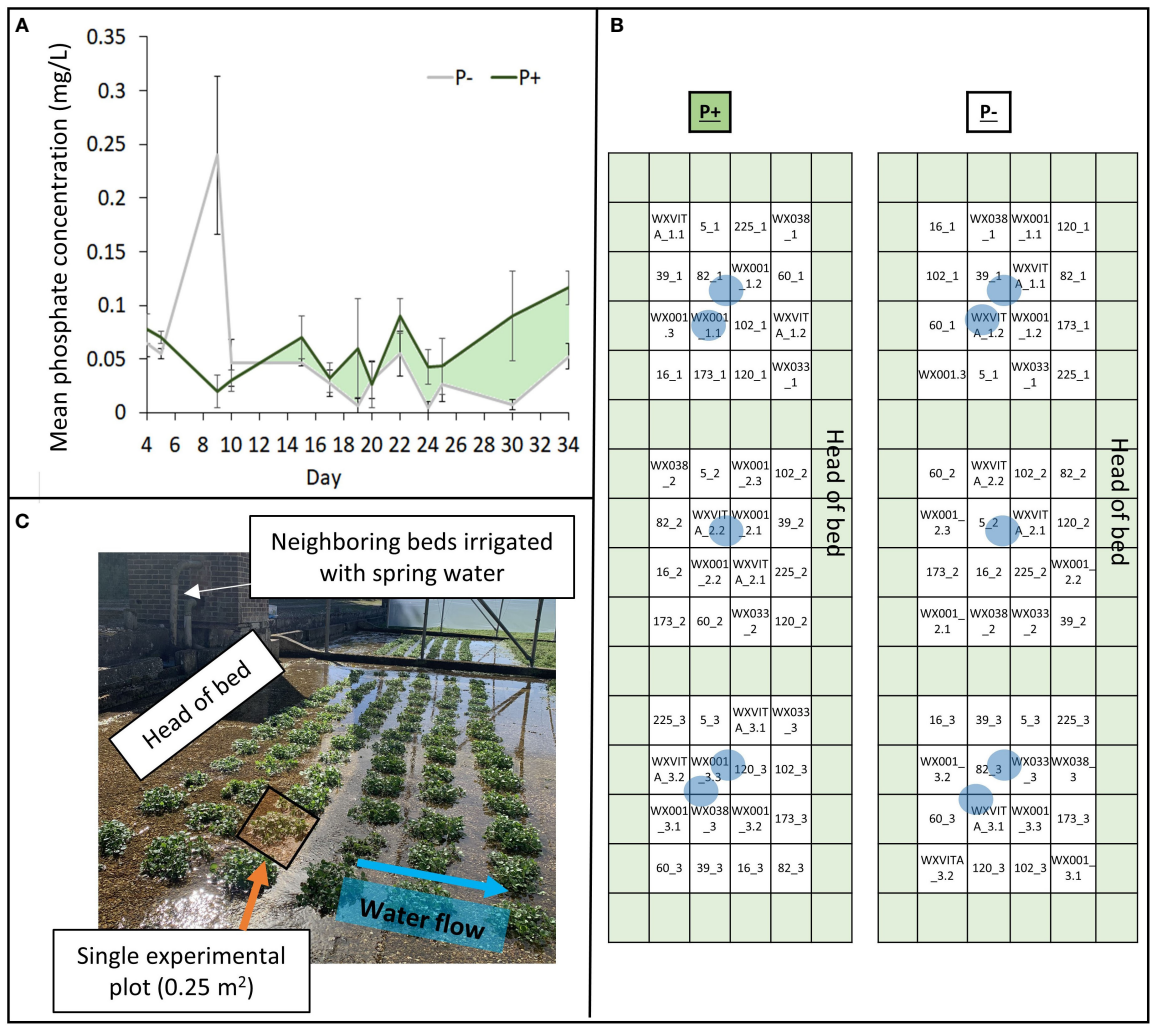
Elements of the field design. **(A)** Phosphate concentrations within treatments (P+/P−). P+ received doses of fertilizer on days 16 and 23, P− was untreated. Areas shaded light green represent periods where concentration was higher in the P+ treatment. **(B)** Field plan showing 13 different lines within a randomized complete block design with 3 blocks. Green represents plots of guard plants and blue circles indicate P determination sampling sites (both within and between plots). **(C)** Images of the field to illustrate plants grouped within 0.25 m^2^ plots. Neighboring beds were irrigated using the same spring water pump and the direction of water flow across the bed is indicated by the blue arrow.

F_2:4_ seeds were sown in peat-filled trays. Tray positioning was randomized in the greenhouse (Vitacress Herbs; Chichester, UK) and irrigated with potable water from an overhead sprinkler four times a day. After 3 weeks, plants were thinned to equal density and transplanted into prepared gravel lined beds utilizing a complete randomized block design at a commercial watercress farm (51°11’42.9”N, 1°32’12.9”W; Hampshire, UK). Blocks were composed of 16 0.25 m^2^ plots comprising 35 plants per plot. Trial areas were located at the heads of neighboring gravel-lined beds, with a shared flowing spring water supply at an almost constant temperature of 12°C (**Figure 1**). Beds were covered by an open-ended polytunnel to reduce bird damage and buffer from environmental variation. One bed was given no additional fertilizer (P−) during the trial period, and the other was supplied with a standard commercial fertilizer regime (P+) as follows: one dose of base dressing (Humber Palmer Eco-Cress Base; details of fertilizer in **Supplementary 1**) on day 16, then one dose of top dressing (Humber Palmer Eco-Cress Plus) on day 23 post-transplanting. The application rates were 200 kg ha^−1^ of base dressing (30.4 kg ha^−1^ P_2_O_5_) and 100 kg ha^−1^ of top dressing (12 kg ha^−1^ P_2_O_5_).

### 2.3 Measuring P concentration of irrigation supply

To monitor P bioavailability during the trial, phosphate in the irrigation supply was quantified using a low range handheld phosphate photometer using an adaption of the ascorbic acid/molybdenum blue method (HI-96713; HANNA instruments). Samples were taken at least every 5 days within the bed (**Figure 1A**), prior to harvesting plants, 24 h before, and 24 h after each fertilizer application to increase data granularity. Eight additional water samples were collected at the final harvest point and were assessed for ammoniacal nitrogen, nitrate (NO_3_), P (total unfiltered), and orthophosphate (PO_4_) concentration by ALS (alsglobal.com) to support handheld phosphate measurements. Though P concentrations fluctuated throughout the trial period in this commercial watercress bed (**Figure 1A**), overall P concentration was higher in the fertilized bed for the majority of measurements. Additional analyses at the final harvest point showed that nitrogen concentration in both beds did not significantly differ (P+ 34.48 mg/L ± 0.15; P− 34.03 mg/L ± 0.54), increasing evidence that P is the limiting macronutrient at the point of harvest.

### 2.4 Phenotyping

#### 2.4.1 Morphological measurements

After 35 days following transplanting, four plants were sampled per plot for morphological analyses. The following traits were recorded: shoot/root fresh and dry weight, stem length (identified as length of the main stem), and number of leaves. Root:shoot (R:S) ratio was calculated from fresh weight values. Images of dissected plants were used to quantify leaf area parameters (mean and total leaf area) on ImageJ software (Schneider et al., 2012). One plant was also selected from each plot every 5 days to assess morphological changes over time. Watercress forms a densely matted root structure under commercial growing conditions; thus, quantifying aspects of root architecture was not possible in this study.

#### 2.4.2 Biochemical measurements: quantifying AO capacity, GSL, sugar, starch, P, and K concentration

After 35 days, one plant was selected from each plot, roots were cleaned and cut from the shoot, and both tissue portions were frozen separately in liquid nitrogen. Frozen tissue was ground to a fine homogeneous powder and stored at −80°C prior to further analyses.

The AO capacity of each sample was assessed using the Ferric Reducing Ability of Plasma (FRAP) protocol as described previously (Benzie and Strain, 1996; Payne et al., 2013; Qian et al., 2023). Ground frozen tissue was transferred to QIAshredder homogenizer tubes (Qiagen), weighed, and spun at 20,000 rpm for 5 min. Extracted sap was transferred to 96-well plates alongside a serial dilution of iron sulfate heptahydrate. FRAP reagent solution, containing acetate buffer, TPTZ (2,4,6-Tri(2-pyridyl)-s-triazine), and iron chloride hexahydrate, was added to the plate and immediately read on a spectrophotometer (CLARIOstar Plus; BMG Labtech) at 620 nm. Plates were run in duplicate.

GSLs were quantified by HPLC-DAD as described by Kliebenstein et al. (2001) and used previously for watercress (Qian et al., 2023). Briefly, 20–40 mg of ground frozen tissue was weighed and homogenized in a paint shaker with 90% methanol for 3 min and centrifuged. Ninety-six-well filter plates were loaded with DEAE Sephadex A-25 and the plant supernatants, then washed with water, 90% methanol, and water again. Following an overnight incubation with sulfatase, the Sephadex-bound GSLs were eluted. Desulfoglucosinolates were separated and detected by HPLC-DAD and quantified by comparison to standard curves of purified compounds and results were normalized to fresh weight.

Soluble (sugar) and insoluble (starch) carbohydrates were determined using a modified anthrone method (Leyva et al., 2008; Becerra-Sanchez and Taylor, 2021). One milliliter of buffer (sodium acetate 0.2 mol/L, pH 5.5) was added to 5–50 mg of pre-weighed lyophilized tissue and incubated at 70°C for 15 min. Samples were centrifuged for 10 min at 15,000 rpm, then 50 µL of supernatant was transferred to fresh tubes containing 1 mL of ultrapure water for sugar quantification. The remaining pellets were vortexed and incubated at 100°C for 10 min. Enzymatic digestion was conducted by adding 100 µL of 70 units/mL amyglucosidase and 100 µL of 7 units/mL alpha amylase and incubating pellets for 2 h at 37°C. Tubes were centrifuged at 15,000 rpm for 10 min and 50 µL of supernatant diluted in 1 mL of ultrapure water for starch determination. Samples were plated into 96-well plates in duplicate, alongside glucose calibration curves. A super-standard of pooled watercress shoot samples was also run alongside each batch of samples to standardize runs. Anthrone in sulfuric acid (150 µL; 0.1% w/v) was added per well and incubated for 20 min at 100°C. Absorbance at 620 nm was analyzed on a Multiskan™ FC Microplate Photometer (Thermo Scientific).

Lyophilized root and shoot tissue was also used for quantification of P and potassium (K) by ICP-MS. Sample digestion and ICP-MS analysis was conducted by the Interdisciplinary Center for Plasma Mass Spectrometry at the University of California Davis using an Agilent 8900 ICP-MS (Agilent Technologies, Palo Alto, CA). Samples, duplicate method blacks (50 µL of 18.2 MΩ/cm water), duplicate digestion quality control standards (50 µL), and duplicate standards from tomato (NIST1573a) and spinach (NIST1570a) were digested. Acid digestion involved adding 0.75 mL of 50% HNO_3_ to samples in two increments, each time allowing gas to evolve and then heating for 35 min and 1 h at 95°C, respectively. After cooling, 50 µL of H_2_O_2_ was added incrementally up to 500 µL as samples were heated, then heated for 1 h after the final addition. Finally, samples were allowed to cool and brought to a final volume of 1 mL with 18.2 MΩ/cm water, ready for analysis. ICP-MS analysis was conducted by the Interdisciplinary Center for Plasma Mass Spectrometry at the University of California Davis using an Agilent 8900 ICP-MS (Agilent Technologies, Palo Alto, CA).

### 2.5 RNA extraction

RNA was extracted from frozen ground root and shoot tissue taken at the final harvest point (35 days post-transplanting) using a modified cetyltrimethylammonium bromide (CTAB) protocol used previously for watercress (Doyle and Doyle, 1987; Payne et al., 2015; Voutsina, 2017). Tissue (200–300 mg) was weighed and incubated with 900 µL of pre-warmed CTAB (+50 µL of 2-mercaptoethanol) at 65°C for 5 min. CHISAM (Chloroform : Isoamyl alcohol 24:1; 800 µL) was added and tubes were spun at 12,000 rpm for 10 min at room temperature. The aqueous phase was transferred to a fresh tube and the CHISAM step was repeated. A total of 180 µL of 10 M LiCl was then added to the aqueous phase before precipitation at 4°C overnight. Samples were spun at 4°C, then the supernatant was discarded, and the pellet was dissolved in 700 µL of pre-warmed SSTE. Tubes were incubated at 60°C for 5 min followed by a repeat of the CHISAM step. The aqueous phase was transferred to a fresh tube and 700 µL of 100% cold ethanol was added. This was left to precipitate at −20°C for 10 min before centrifugation and removal of supernatant. The remaining pellet was washed with cold 70% ethanol, left to air dry (>45 min) and redissolved in 50 µL of RNase-free H_2_O.

### 2.6 RNA-sequencing data analysis pipeline

RNA-seq data for both roots and shoots were processed using a pipeline adapted from that used previously for watercress (Qian, 2021). Novogene Corporation Inc. (Sacramento) provided the eukaryotic RNA-seq service including cDNA library preparation (250–300 bp insert) and sequencing using the Illumina HiSeq (paired-end 150 bp) platform. Raw data processing was conducted using the UC Davis Farm cluster (https://www.hpc.ucdavis.edu/farm-cluster) followed by differential gene expression analysis in R Studio. To check FASTQ file quality, FastQC and MultiQC were conducted. The Trimmomatic preprocessing tool designed to handle paired-end Illumina sequence data was used to trim and remove poor-quality reads (Bolger et al., 2014). The first whole genome sequencing and assembly of the watercress genome has been completed by IGATech (https://igatechnology.com) and provided to the laboratory of Prof. Gail Taylor. Functional genome annotation was done using Interproscan 5.0. and by searching the scanned protein sequence against the UniProt database (https://www.uniprot.org). The genome file was indexed to improve the efficiency of searching the genome using the Hierarchical Graph FM index (HGFM) with the alignment program HISAT2. HISAT2 was also used to align reads to the watercress genome. Then, StringTie was used to assemble read alignments into potential transcripts, and the output was used to generate a count table with featureCounts (Liao et al., 2014; Pertea et al., 2016).

Differential gene expression analysis was conducted in R using the edgeR package using a GLM approach (Robinson et al., 2010). Raw counts were filtered to include only genes with 1 count per million (cpm) in at least two samples and library sizes were normalized. Quasi-likelihood *F*-tests were performed to provide more robust and reliable error rate control for smaller replicate numbers. Significance testing was conducted using the Benjamini–Hochberg method and differentially expressed genes (DEGs) were selected with a cutoff at FDR < 0.05 (Benjamini and Hochberg, 1995). Finally, gene ontology (GO) enrichment analysis was conducted on DEG lists using ShinyGO with *A. thaliana* as a reference and Venn diagrams were generated using the online tool VENNY v2.1 (Oliveros, 2015; Ge et al., 2020).

### 2.7 Statistical analyses of morphological and biochemical data

SPSS (version 27, IBM Corp, 2020) and R software (R Core Team, 2021) were used for statistical analyses. Results were averaged per plot to get the genotypic mean per block (*n* = 3). Normality of residuals was assessed using Q–Q plots and Shapiro–Wilk tests, and homogeneity of variances was checked using Levene’s test of equal variances. Linear mixed effect models were generated with block as a random factor and line and treatment as fixed main effects. Analysis of variance (ANOVA) tests were run on these models to identify differences between treatment and line with a significance threshold of *p* < 0.05.

## 3 Results

### 3.1 Effect of fertilizer application on watercress morphology

**Figure 2:**
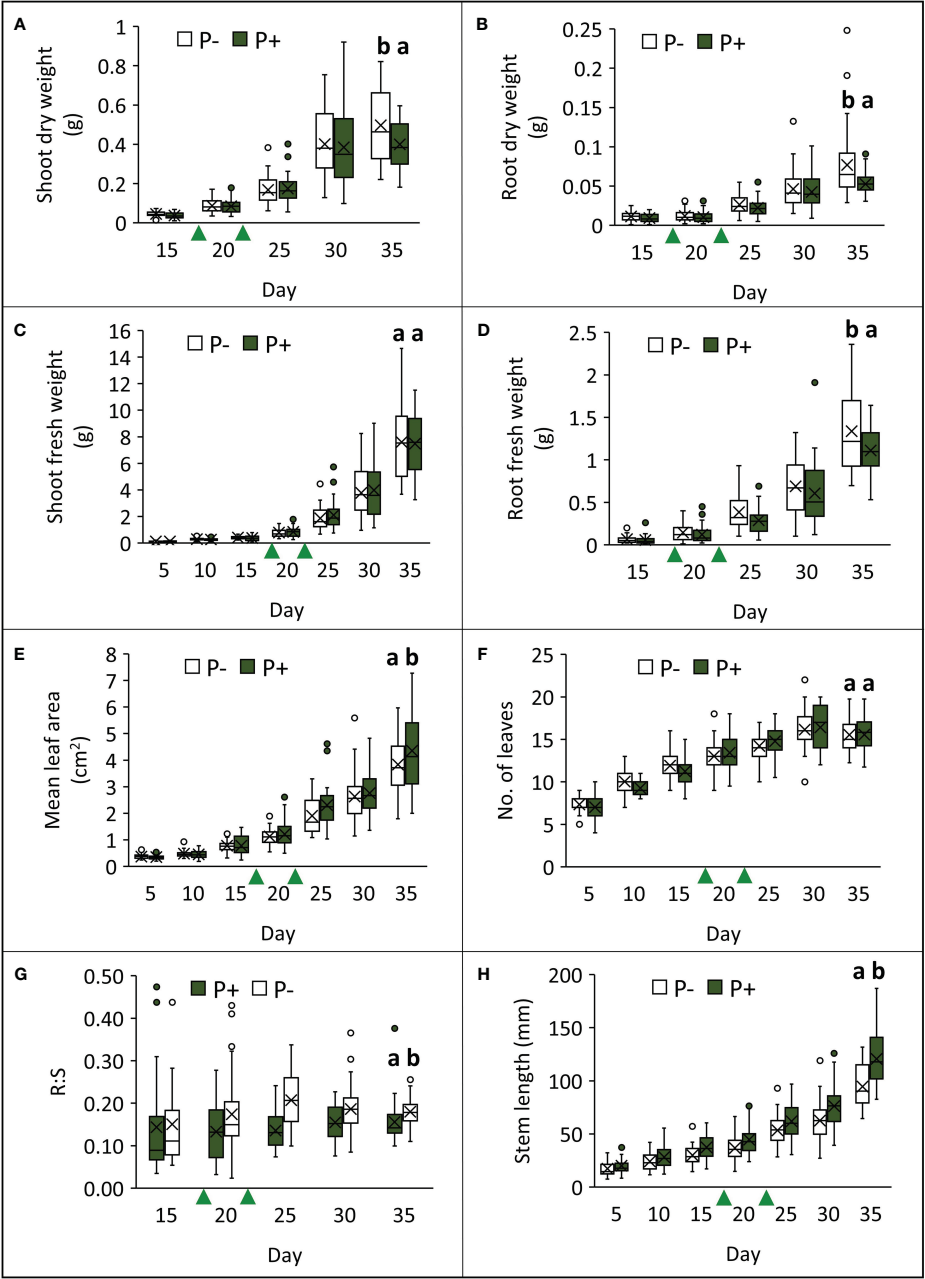
Effects of phosphate-based fertilizer application (P+) on different aspects of watercress morphology **(A–H)**, compared to plants grown without additional phosphate-based fertilizer (P−) over the course of the field trial. Green triangles indicate when P treatments were applied. Crosses within bars denote mean values (*n* = 39) and letters above bars represent different groups according to Tukey’s LSD tests conducted on data from the final harvest point (*p* < 0.05).

Growing watercress in P− conditions impacted several morphological parameters at final harvest (day 35; **Table 1**; **Figure 2**; **Supplementary 2**). The removal of fertilizer application resulted in a 20% increase in root fresh weight (*F*_1,49_ = 7.942, *p* = 0.007), a 46% increase in root dry weight (*F*_1,49_ = 12.040, *p* = 0.001), and a 24% increase in shoot dry weight (*F*_1,49_ = 8.819, *p* = 0.005). However, shoot fresh weight was not affected by treatment (shoot: *F*_1,49_ = 0.055, *p* = 0.815; total: *F*_1,49_ = 0.260, *p* = 0.612). Stem length decreased by approximately 22% in P− (*F*_1,49_ = 36.803, *p* < 0.001). Together, this is reflected in a ~24% increase in root:shoot ratio in P− (*F*_1,48_ = 28.340, *p* < 0.001). Although, the number of leaves (*F*_1,49_ < 0.001, *p* = 0.981) did not change between treatments, mean leaf area decreased by 12% (*F*_1,49_ = 5.864, *p* = 0.019).

**Table 1:** Effect of phosphate-based fertilizer application on morphology and biochemistry of watercress at the final harvest point.

Trait	P+	P−	Treatment	Line	T*L
Shoot dry weight (g)	0.40 (± 0.02)	0.50 (± 0.03)	**	ns	ns
Root dry weight (g)	0.05 (± 0.002)	0.077 (± 0.007)	**	ns	ns
Shoot fresh weight (g)	7.47 (± 0.35)	7.60 (± 0.45)	ns	ns	ns
Root fresh weight (g)	1.11 (± 0.04)	1.34 (± 0.08)	**	ns	ns
Root:shoot	0.15 (± 0.005)	0.19 (± 0.005)	***	ns	ns
Stem length (mm)	120.56 (± 3.84)	94.52 (± 3.03)	***	**	ns
No. of leaves	15.55 (± 0.32)	15.53 (± 0.32)	ns	***	ns
Individual leaf area (cm^2^)	4.35 (± 0.23)	3.83 (± 0.17)	*	***	ns
AO capacity (mmol Fe^2+^ per g FW)	429.41 (± 17.39)	485.49 (± 14.96)	*	ns	ns
GSL concentration (nmol/mg FW)	0.59 (± 0.036)	0.62 (± 0.050)	ns	ns	ns
Sugar concentration (mg/g DW)	107.27 (± 2.50)	118.43 (± 3.44)	*	ns	ns
Starch concentration (mg/g DW)	25.86 (± 2.51)	43.07 (± 3.30)	***	ns	ns
Mean phosphorus concentration (ppm)	3,619 (± 196)	2,801 (± 142)	***	*	ns

Mean values ± SEM are given for each treatment (P+/P−) alongside main effects given at the following significance levels: *p < 0.05, **p < 0.01, ***p < 0.001, ns non-significant. Phosphorus concentration was calculated as the mean of shoot and root values from selected lines. T*L, treatment*line interaction effect.

### 3.2 P-fertilizer application alters the biochemical profile

**Figure 3:**
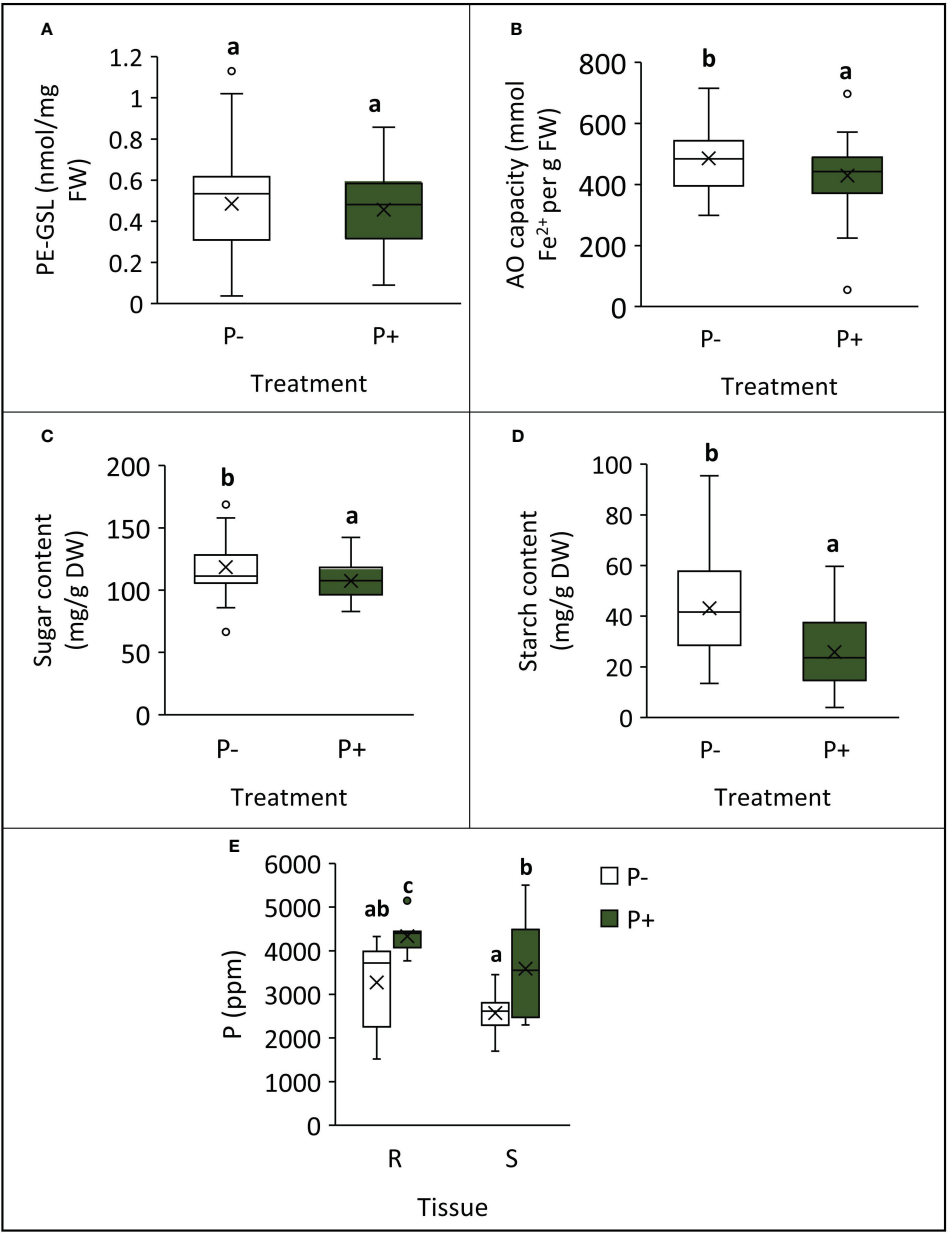
Differences in biochemical traits at final harvest point, following cultivation with (P+) or without (P−) phosphate fertilizer. **(A)** PE-glucosinolate (PE-GSL) concentration of shoots; **(B)** antioxidant capacity of shoots; **(C)** shoot sugar concentration; **(D) **shoot starch concentration; **(E)** phosphorus concentration in roots (R) and shoots (S) from selected lines. Means are denoted by crosses within boxes (**A–D**: *n* = 39; **E**: *n* = 9) and letters represent significantly different groups by Tukey’s LSD tests (*p* < 0.05).

Plants grown without additional phosphate-based fertilizer exhibited changes to their biochemical profile at harvest point (**Table 1**; **Figure 3**). Shoot AO capacity increased by 13% (*F*_1,49_ = 6.090, *p* = 0.017). When considering the concentration of the primary GSL in watercress, PE-GSL (phenylethyl glucosinolate), there was no effect of fertilizer treatment (*F*_1,49_ = 0.363, *p* = 0.550). The concentration of soluble sugars increased by 10% under P− (*F*_1,49_ = 6.957, *p* = 0.011) and starch increased by 67% (*F*_1,49_ = 23.835, *p* < 0.001). The concentration of P and K was assessed in selected lines (WXVITA, 102, 60; line selection is described in the subsequent section). As expected, P concentration of both roots and shoots increased in the P+ treatment (*F*_1,17_ = 17.586, *p* < 0.001): root P concentration increased from 3,274 ppm ( ± 352) to 4,333 ppm ( ± 128), and shoot P concentration increased from 2,575 ppm ( ± 160) to 3,586 ppm ( ± 366), whereas K concentration was unaffected by treatment (*F*_1,17_ = 1.19, *p* = 0.34), providing further evidence that P is the limiting macronutrient in this trial.

### 3.3 Morphological and biochemical data support significant line differences for future selection

**Figure 4:**
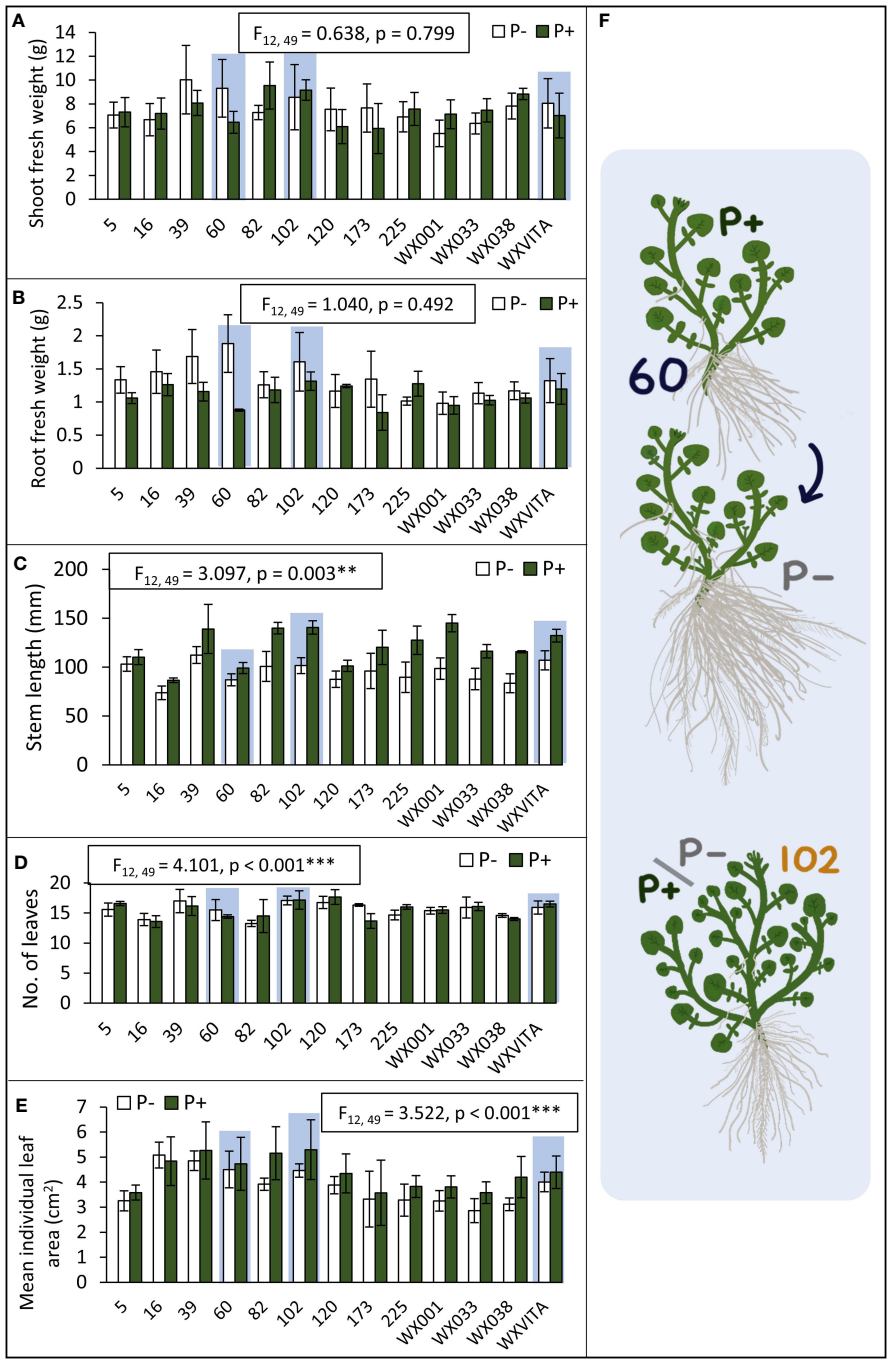
Genetic variation between lines to inform line selection **(A–E)**. Lines that were selected for RNA-seq (60, 102, and WXVITA) are highlighted in light blue. Error bars represent SEM. Statistics for the effect of line on each trait (from ANOVA tests) are given in text boxes. Line effects are significant at the following levels: **p < 0.01, ***p < 0.001. **(F)** Illustrations of representative plants from selected lines. Line 60 is P-responsive with a large increase in root biomass in P−, whereas 102 maintains high yield under both P conditions.

**Figure 5:**
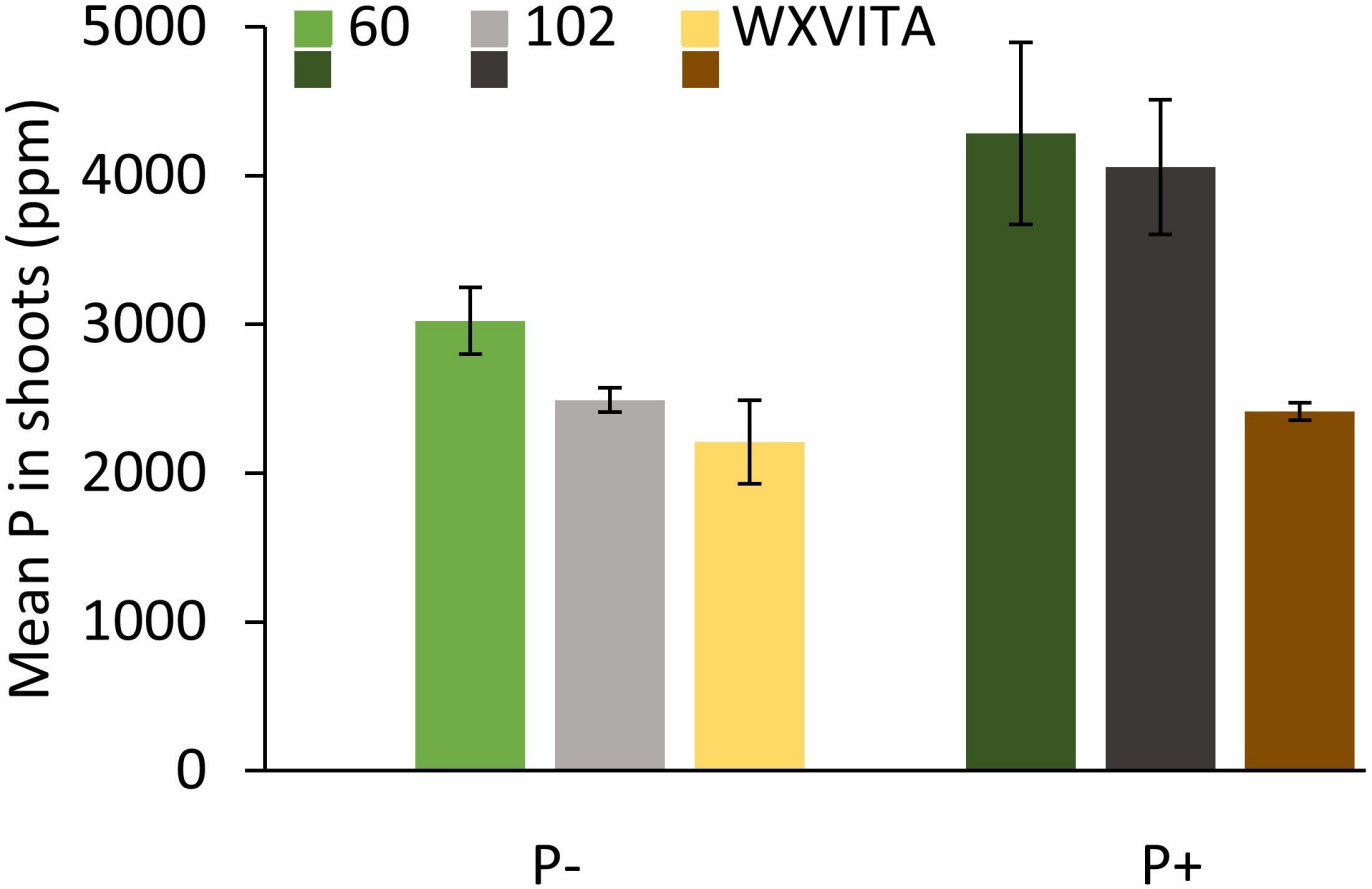
Total phosphorus (P) concentration in dried shoot tissue of selected watercress lines (60, 102, and WXVITA) at final harvest. Error bars are representative of SEM (*n* = 3).

For each of the 13 lines studied, the following morphological and biochemical traits were quantified: shoot dry weight, root dry weight, shoot fresh weight, R:S, stem length, no. of leaves, individual leaf area, AO capacity, GSL concentration, sugar concentration, and starch concentration. Of these traits, lines varied in stem length (*F*_12,49_ = 3.100, *p* = 0.003), no. of leaves (*F*_12,49_ = 4.101, *p* < 0.001), and mean leaf area (*F*_12,49_ = 3.522, *p* < 0.001; **Figure 4**). Statistics and figures showing line variation for all other traits are provided in **Supplementary 3**. To quantify responsiveness to low P conditions, percentage changes under P− conditions were calculated for traits. Line 60 showed a 35% change across all morphology traits under P−, including a 114% increase in mean root fresh weight, suggesting that it is highly responsive to low nutrient conditions. This line is also of note as it ranked highest for root dry weight and had the third highest AO capacity. For other traits such as leaf area and shoot fresh weight, it consistently ranked in the top half. Line 102 is also of interest as it ranked highest for several commercially relevant yield traits such as shoot fresh weight, second highest for no. of leaves, and third highest for PE-GSL concentration. By contrast, this line was far less responsive to P− conditions: there was a 15.5% change across all morphology traits and only a 0.9% increase and 6.6% decrease in shoot dry and fresh weight, respectively. These lines were taken forward for further analysis of P concentration and for RNA-seq analysis. P concentration also differed between these lines (*F*_2,17_ = 4.446, *p* = 0.028; **Figure 5**). Comparing across treatments, shoot P concentration was 42% higher in 102 compared to the commercial control line WXVITA and line 60 had 58% increase relative to WXVITA (*t*_6_ = 3.878, *p* = 0.019).

### 3.4 RNA-seq identifies genes important for P-regulation

Between 29 and 43 million reads per library were produced from RNA-seq with all libraries having 70%–91% of reads mapping uniquely to the watercress genome. A total of 44,024 transcripts were identified, and of these, 27,149 were represented at least 1 cpm in at least two shoot samples, and 27,531 were represented at least 1 cpm in at least two root samples. Eighty-eight percent of shoot and root transcripts corresponded to annotated watercress genes. No DEGs were detected in P− shoots compared to P+ shoots (FDR < 0.05). However, 16 genes had an FDR < 0.15 with corresponding *p*-values < 1e-5 (**Supplementary 4**). This list included a 0.55- and 1.7-fold upregulation in *GDPD5* (AT1G74210) and *GDPD1* (AT3G02040), involved in lipid remodeling during P deficiency (Cheng et al., 2011). Genes involved in carbohydrate metabolism, such as *CTIMC* (AT3G55440) and the phosphoglucan phosphatase *SEX4* (AT3G52180), were upregulated in P− conditions (Kötting et al., 2009). Upregulation of *VTC4* (AT3G02870) is notable as it encodes a bi-functional enzyme involved in myoinositol and ascorbate synthesis (Torabinejad et al., 2009). Myoinositol has roles for P signaling, storage, and stress tolerance, and ascorbate regulates several abiotic stress signaling pathways and provides AO functions (Xiao et al., 2021; Wu et al., 2023). *PLAT1* (AT4G39730), involved in a broad range of abiotic stress response pathways, was also upregulated in P− (Hyun et al., 2014).

**Figure 6:**
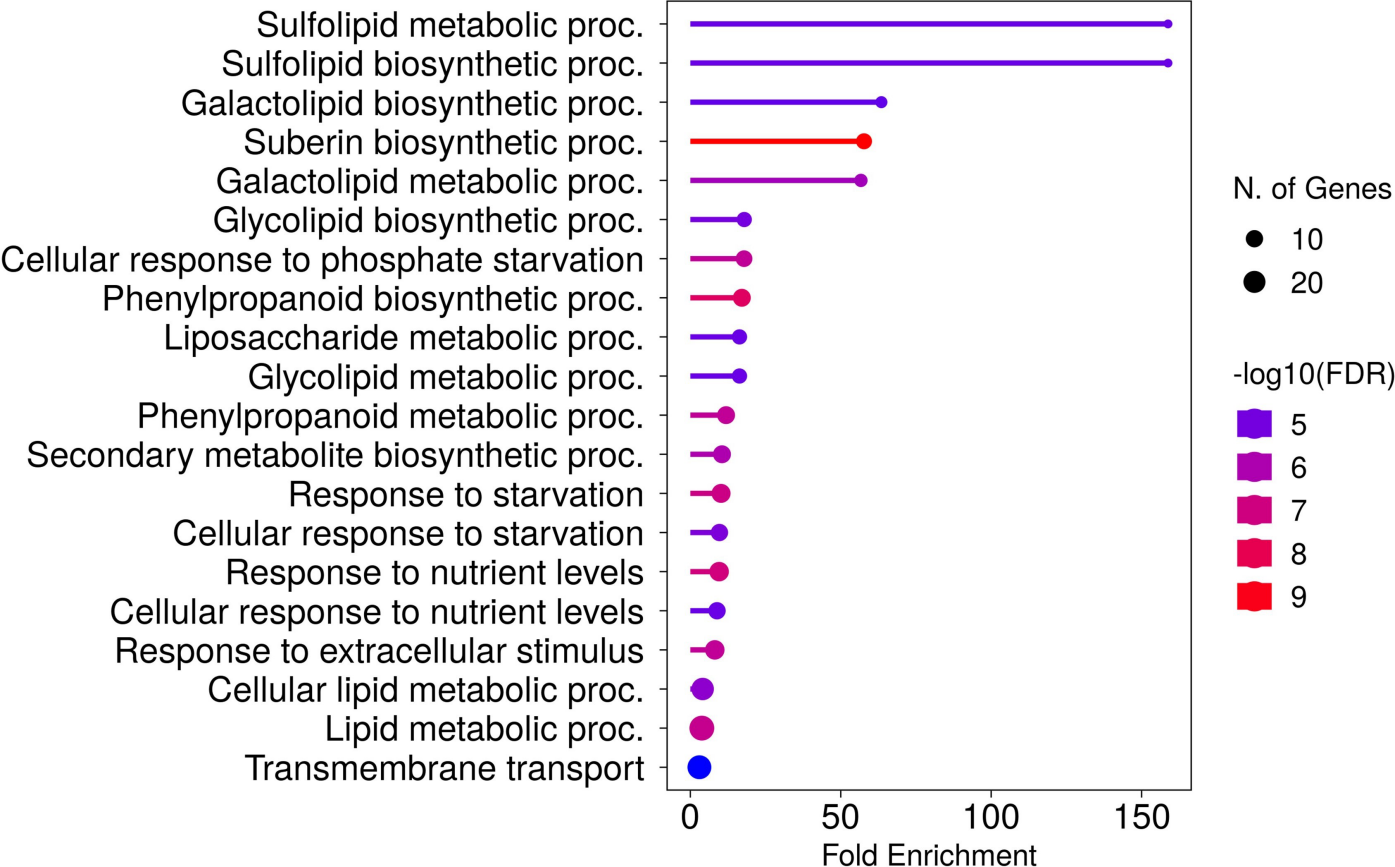
Top 20 enriched biological processes of DEGs in roots of watercress grown without additional phosphate-based fertilizer. Fold enrichment is defined as the percentage of expressed sequences of known annotation belonging to a pathway, divided by the corresponding percentage in the background. Developed using the ShinyGO platform with *A. thaliana* as a reference.

In roots, 33 genes were significantly downregulated and 227 genes were significantly upregulated in P− watercress roots with respect to the P+ treatment. Of these, 173 were annotated and almost all annotated genes corresponded to those in *A. thaliana*. The top 50 annotated DEGs are summarized in **Table 2**, and the full list of DEGs is shown in **Supplementary 5**. GO analysis (**Figure 6**) revealed significant enrichment of pathways involved in known responses to P deficiency. The highest enrichment was observed for genes involved in sulfolipid metabolic and biosynthetic processes (>158-fold enrichment) through upregulation of *SQD1* (AT4G33030), *SQD2* (AT5G01220), and *UGP3* (AT3G56040), followed by a 63-fold enrichment of genes involved in galactolipid biosynthesis including *DGD1* (AT3G11670), DGD2 (AT4G00550), *MGD3* (AT2G11810), *PAH1* (AT3G09560), and *PLPZETA2* (AT3G05630) (Yu et al., 2002; Cruz-Ramirez et al., 2006; Kobayashi et al., 2009). Two upregulated genes, *PHT1;8* and *PHT1;4*, encode high-affinity phosphate transporters in *A. thaliana* (Nussaume et al., 2011). Other genes associated with a cellular response to P starvation included *RNS1* (AT2G02990) for P remobilization, and *SPX1* (AT5G20150) and *SPX3* (AT2G45130), which are essential transcriptional regulators of the P-starvation response (Puga et al., 2014; Zhou et al., 2015).

**Table 2:** List of the top 50 annotated DEGs in P− roots relative to P+ roots.

Transcript ID	logFC	*p*-value	FDR	Annotation	*At* identifier	Function	Reference
MSTRG.23989	0.95	4.9E-08	9.20E-04	ARFI_ARATH	AT4G23980	Transcription factor.	(Ulmasov et al., 1997)
**MSTRG.14858**	**1.92**	**1E-07**	**9.20E-04**	**SPX3_ARATH**	**AT2G45130**	**Positive role in plant adaptation to P starvation.**	(Duan et al., 2008)
MSTRG.14179	1.01	2.48E-07	1.71E-03	PER20_ARATH	AT2G35380	Suberization, response to oxidative stress.	(Welinder et al., 2002; Wan et al., 2021)
MSTRG.35586	0.94	1E-06	2.59E-03	BDG3_ARATH	AT4G24140	Involved in cuticle development and morphogenesis (based on protein similarity).	(Kurdyukov et al., 2006)
MSTRG.16022	1.12	1.03E-06	2.59E-03	CSPLW_ARATH	AT5G44550	Uncharacterized protein family. Highly expressed in root (Casparian strip).	(Roppolo et al., 2014)
**MSTRG.28218**	**0.96**	**1.03E-06**	**2.59E-03**	**PHT18_ARATH**	**AT1G20860**	**PHT1;8 High-affinity P transporter.**	(Remy et al., 2012; Lapis-Gaza et al., 2014)
**MSTRG.32112**	**2.78**	**9.26E-07**	**2.59E-0**3	**PPSP2_ARATH**	**AT1G17710**	**PECP1; phospholipid hydrolysis**	(May et al., 2012; Hanchi et al., 2018; Tannert et al., 2018)
MSTRG.22076	0.65	1.01E-06	2.59E-03	STY8_ARATH	AT2G17700	Serine/threonine protein kinase	(Martin et al., 2006)
**MSTRG.7113**	**1.10**	**1.8E-06**	**3.82E-03**	**MGDG3_ARATH**	**AT2G11810**	**Membrane lipid remodeling in P-starved roots.**	(Kobayashi et al., 2009)
MSTRG.16576	0.56	3E-06	3.85E-03	ADH1_ARATH	AT1G77120	Alcohol dehydrogenase, response to hypoxia	(Ismond et al., 2003)
MSTRG.20017	1.13	3E-06	3.85E-03	GDL38_ARATH	AT2G23540	May catalyze acyltransfer or hydrolase reactions.	(Akoh et al., 2004)
MSTRG.33131	0.64	2.73E-06	3.85E-03	KCS1_ARATH	AT1G01120	Contributes to cuticular wax and suberin biosynthesis.	(Todd et al., 1999)
MSTRG.10623	1.06	3.21E-06	3.85E-03	LTG13_ARATH	AT2G44290	Non-specific lipid transfer protein GPI-anchored 13	(Borner et al., 2003; Edstam et al., 2013)
MSTRG.15471	1.22	2.66E-06	3.85E-03	NAT4_ARATH	AT1G49960	Xanthine/uracil permease family protein	(Maurino et al., 2006)
**MSTRG.30965**	**0.98**	**2.83E-06**	**3.85E-03**	**SQD2_ARATH**	**AT5G01220**	**Sulfolipid biosynthesis. For substitute of phospholipids under P-deficiency.**	(Yu et al., 2002)
**MSTRG.9588**	**0.61**	**3.65E-06**	**4.18E-03**	**DGDG1_ARATH**	**AT3G11670**	**Involved in the synthesis of diacylglycerol galactolipids, part of P-starvation response.**	(Dörmann et al., 1999)
MSTRG.32990	0.90	4.81E-06	4.76E-03	ATL1_ARATH	AT1G04360	RING/U-box superfamily protein	(Serrano et al., 2014; Jiménez-López et al., 2018)
MSTRG.8205	1.15	4.84E-06	4.76E-03	GPAT5_ARATH	AT3G11430	Involved in suberin and phospholipid metabolism	(Beisson et al., 2007)
MSTRG.26237	0.56	4.72E-06	4.76E-03	PAS1_ARATH	AT3G54010	Essential protein regulating cell division, adhesion, and elongation.	(Faure et al., 1998; Harrar et al., 2003; Smyczynski et al., 2006)
MSTRG.18843	0.96	4.5E-06	4.76E-03	PER11_ARATH	AT1G68850	Biosynthesis and degradation of lignin, suberization, auxin catabolism, and response to environmental stresses.	(Tognolli et al., 2002; Yu et al., 2016)
MSTRG.12851	1.08	5.85E-06	5.55E-03	C86A1_ARATH	AT5G58860	CYP86A1; Catalyzes the omega-hydroxylation of fatty acids.	(Benveniste et al., 1998; Duan and Schuler, 2005)
**MSTRG.34984**	**1.01**	**7.18E-06**	**6.37E-03**	**SQD1_ARATH**	**AT4G33030**	**Sulfolipid biosynthesis. Can function as a substitute of phospholipids under P-deficiency**	(Essigmann et al., 1998; Sanda et al., 2001)
MSTRG.15440	0.53	7.88E-06	6.58E-03	LACS2_ARATH	AT1G49430	Lipid metabolism, required for repression of lateral root formation.	(Schnurr et al., 2004; MacGregor et al., 2008)
MSTRG.34535	1.09	8.93E-06	7.23E-03	CP18C_ARATH	AT4G38740	Involved in hypersensitive response and plant defense.	(Romano et al., 2004; Coaker et al., 2005)
MSTRG.3173	0.89	9.84E-06	7.32E-03	AB6G_ARATH	AT5G13580	ABCG6; transporter that is required for synthesis of an effective suberin barrier in roots	(Verrier et al., 2008; Yadav et al., 2014)
MSTRG.35761	0.48	9.62E-06	7.32E-03	FHYRK_ARATH	AT4G21470	Enzyme that catalyzes the hydrolysis of flavin-mononucleotide (FMN) to riboflavin, and the phosphorylation of riboflavin to FMN.	(Sandoval and Roje, 2005)
MSTRG.35999	0.50	9.46E-06	7.32E-03	FLZ2_ARATH	AT4G17670	Involved in response to sugars, response to starvation.	(Jamsheer K and Laxmi, 2015)
MSTRG.29846	0.70	1.19E-05	8.04E-03	TSJT1_TOBAC	NA	Function unknown	N/A
**MSTRG.18544**	**1.43**	**1.24E-05**	**8.15E-03**	**OCT1_ARATH**	**AT1G73220**	**High-affinity carnitine uptake transporter. Regulates lateral root development. P-starvation inducible.**	(Lelandais-Brière et al., 2007; Lan et al., 2015)
MSTRG.16770	1.09	1.35E-05	8.65E-03	GDL31_ARATH	AT1G74460	GDSL-motif esterase/acyltransferase/lipase.	(Akoh et al., 2004)
MSTRG.21670	0.39	1.42E-05	8.87E-03	HIS1A_ARATH	AT1G58080	ATP phosphoribosyl transferase, catalyzes first step of histidine biosynthesis	(Ohta et al., 2000)
**MSTRG.8570**	**1.05**	**1.56E-05**	**8.93E-03**	**PLDZ2_ARATH**	**AT3G05630**	**Hydrolyzes phospholipids to generate phosphatidic acids (PA) for galactolipid synthesis in P-starved roots. Involved in root elongation during P limitation.**	(Qin and Wang, 2002; Cruz-Ramirez et al., 2006)
MSTRG.20521	−0.65	1.53E-05	8.93E-03	TPPF_ARATH	AT4G12430	Produce free trehalose. Trehalose accumulation in plant may improve abiotic stress tolerance (based on sequence similarity).	(Schluepmann et al., 2004)
MSTRG.12401	0.90	1.64E-05	9.22E-03	MYB53_ARATH	AT5G65230	Transcription factor (by similarity)	(Riechmann et al., 2000)
MSTRG.3023	1.05	1.83E-05	9.86E-03	SPSA2_ARATH	AT5G11110	Encodes a sucrose-phosphate synthase. Plays a role in sucrose biosynthesis.	(Lutfiyya et al., 2007; Volkert et al., 2014)
MSTRG.18272	0.48	1.97E-05	9.86E-03	ACR3_ARATH	AT1G76990	Encodes ACT domain-containing protein.	(Hsieh and Goodman, 2002)
MSTRG.33002	1.22	2.01E-05	9.86E-03	KCS2_ARATH	AT1G04220	Involved with suberin biosynthesis pathway	(Joubès et al., 2008; Lee et al., 2009)
MSTRG.36159	0.50	1.95E-05	9.86E-03	WTR33_ARATH	AT4G15540	Nodulin MtN21-like transporter family protein. The mRNA is cell-to-cell mobile.	(Thieme et al., 2015)
MSTRG.21018	0.91	2E-05	9.86E-03	Y5285_ARATH	AT5G14285	DNA-binding storekeeper (inferred)	N/A
MSTRG.29610	1.26	2.08E-05	1.00E-02	C86B1_ARATH	AT5G23190	Involved in very long chain fatty acids (VLCFA) omega-hydroxylation.	(Compagnon et al., 2009)
MSTRG.11422	0.55	2.18E-05	1.02E-02	CALSA_ARATH	AT2G36850	Involved in sporophytic and gametophytic development.	(Töller et al., 2008; Chen et al., 2009; Saatian et al., 2018)
MSTRG.13894	−2.06	2.21E-05	1.02E-02	CLE6_ARATH	AT2G31085	Extracellular signal peptide that regulates cell fate.	(Strabala et al., 2006)
MSTRG.29774	0.89	2.27E-05	1.02E-02	HBPL1_ARATH	AT3G10130	Heme-binding-like protein	(Shanmugabalaji et al., 2020)
MSTRG.30034	0.81	2.38E-05	1.02E-02	GLN14_ARATH	AT5G16570	Homeostatic control of glutamine synthesis in roots	(Ishiyama et al., 2004)
MSTRG.18798	0.66	2.37E-05	1.02E-02	PHL8_ARATH	AT1G69580	Myb family transcription factor.	(Riechmann et al., 2000)
MSTRG.12418	−0.38	2.56E-05	1.03E-02	ANXD2_ARATH	AT5G65020	ANN2; role in regulating root calcium signatures and sugar transport. Also involved in primary root development.	(Wang et al., 2018; Liu et al., 2021)
MSTRG.5527	0.82	2.45E-05	1.03E-02	FRO8_ARATH	AT5G50160	Encodes a ferric chelate reductase	(Wu et al., 2005)
**MSTRG.18862**	**0.81**	**2.49E-05**	**1.03E-02**	**HHO2_ARATH**	**AT1G68670**	**Roles in lateral root development, P mobilization and expression of genes involved in P sensing and signaling.**	(Nagarajan et al., 2016)

MSTRG transcript names from Stringtie output with corresponding annotations (UniProt identifiers) and respective locus identifiers from *A. thaliana* (At) are given. Those in bold have known roles in P-starvation responses in At. N/A, none available.

### 3.5 Genetic differences underlying P-responses between lines

**Figure 7:**
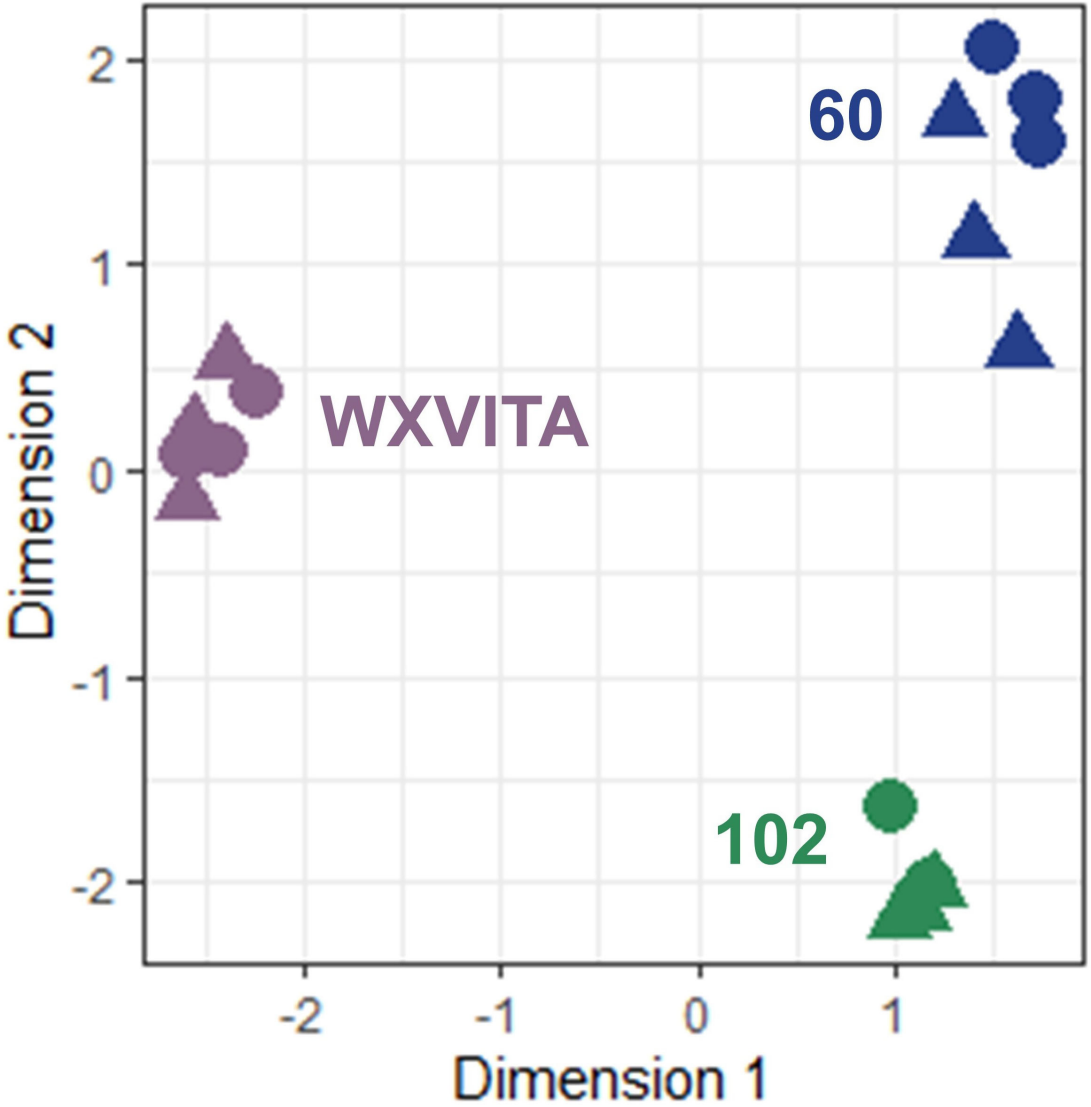
Multidimensional scaling (MDS) of RNA-seq shoot samples. Distances correspond to leading log-fold changes between samples. Circles (◍) and triangles (▲) denote P− and P+ samples, respectively.

**Figure 8:**
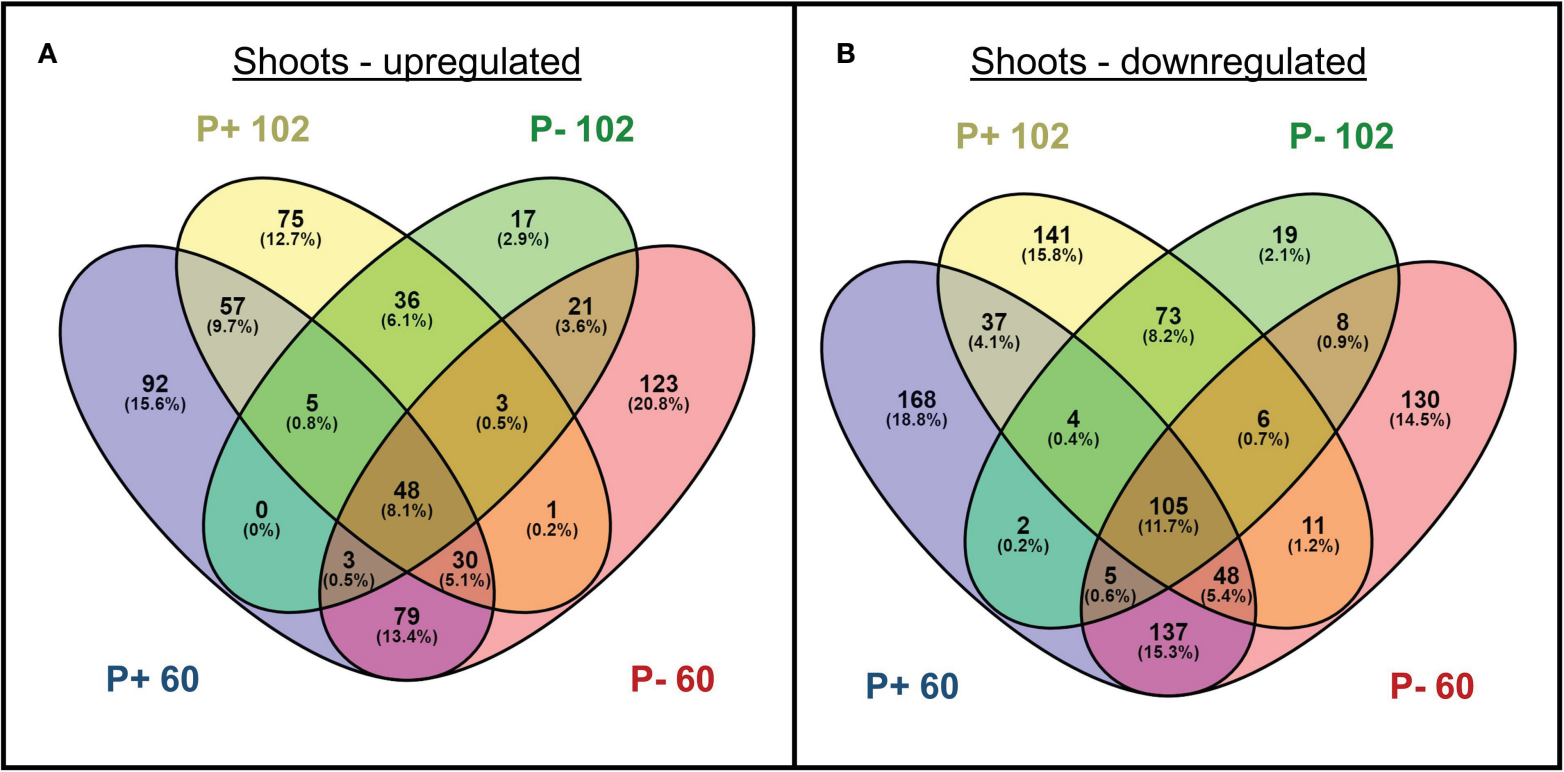
Number of genes **(A)** upregulated and **(B)** downregulated in shoots of selected lines (102 and 60) grown with phosphate-based fertilizer (P+) or without additional fertilizer (P−). Differentially expressed genes are respective to expression in WXVITA. Numbers in parentheses indicate the percentage of DEGs from each group falling into each category.

Multi-dimensional scaling illustrated large transcriptomic differences between line 102, 60, and WXVITA, as shown by differential grouping in **Figure 7**. To assess responses to P-deficiency unique to the selected lines (60 and 102), DEGs in these lines compared to the commercial control line (WXVITA) were identified in P− conditions (**Figure 8**; **Supplementary 6**, **7**). A total of 123 DEGs were uniquely upregulated and 130 DEGs were uniquely downregulated in line 60 shoots in P−, relative to WXVITA. Upregulated genes in line 60 included many genes involved in shoot development, such as *UGE4* (AT1G64440), *MYO6* (AT5G43900), and *PSP* (AT1G18640). By contrast, 17 were uniquely upregulated and 19 were uniquely downregulated in 102 in P− relative to WXVITA. A total of 21 upregulated and 8 downregulated genes were common to both line 60 and 102 relative to expression in WXVITA. GO enrichment analysis of DEGs common to both selected lines identified that the pathway with highest fold enrichment was phosphate ion homeostasis (**Supplementary 8**; 162 fold enrichment), due to upregulation of *CAX1* (AT2G38170) and downregulation of *UBC24* (aka *PHO2*, AT2G33770) (Liu et al., 2011; Liu et al., 2012).

## 4 Discussion

Despite clear environmental issues associated with the leakage of phosphate-based fertilizers into aquatic systems, research targeting breeding for PUE in aquatic crops such as watercress is limited, especially in commercial systems (Austin, 1966; Howard-Williams et al., 1982; Bennett, 1986; Fernandez-Going et al., 2013; Hibbert and Taylor, 2022). In this study, we provide the first comprehensive analysis on the effects of reduced phosphate-based fertilizer supply on watercress morphology, biochemistry, and the transcriptome, and identified novel candidate genes for targeting in future breeding and also new lines with improved tolerance to a low fertilizer cultivation system. These new lines have the potential to be considered as future commercial releases, once these traits are confirmed as fixed.

### 4.1 Watercress root morphology relies heavily on adventitious root growth

Watercress root morphology has previously been shown to be made up of adventitious roots and basal roots (defined as a finer root system that anchors the plant and originates from the primary root meristem) (Cumbus and Robinson, 1977). Of these, the adventitious root system absorbs a greater proportion of P than the basal system and here we found that the watercress root systems in this commercial growth environment form a densely matted structure composed almost completely of roots deriving from the stem (adventitious roots; **Supplementary 9**) at harvest point, suggesting that adventitious roots are most important for P uptake in watercress commercial cultivation and for identifying targets for future watercress selection and breeding.

Growing watercress without additional phosphate-based fertilizer had a significant impact on morphology: mean root biomass was increased, while shoot biomass was either unchanged (fresh biomass) or somewhat increased (dry biomass). Plant stems were shorter with smaller leaves, although the number of leaves was maintained, suggesting that shoot biomass is concentrated in a denser plant. P deprivation results in decreased cell size and is associated with regulation of cell-wall-related genes that cause cellulose synthesis and lignin deposition (Fu et al., 2013; Hoehenwarter et al., 2016; Ogden et al., 2018). The resulting thick-walled small-celled phenotype may explain the dense dwarf shoot phenotype and higher dry mass observed in P−. Most of the evidence for low P-induced cell wall thickening has been investigated only in roots; however, studies in rice have found that plants impaired in the *OsGLU3* gene (associated with cellulose content) exhibit reduced shoot cell growth that is P-dependent (Zhou et al., 2006; Zhang et al., 2012). Three endoglucanases involved in hydrolysis of cellulose (AT1G64390, AT1G75680, and AT2G32990) were downregulated in line 60, P− shoots (compared to WXVITA) in our study, suggesting that this cell-wall thickening response is stronger in this line (Libertini et al., 2004). Although it is surprising that watercress was able to maintain shoot fresh biomass, given the lower availability of P, the increase in root biomass suggests that watercress compensates for reduced nutrient availability by investing more in root growth, particularly for line 60, reflected in the increased R:S ratio in P−, which is a common phenomenon observed in low P conditions (Cogliatti and Clarkson, 1983; Yang et al., 2011; Shen et al., 2018; de Souza Campos et al., 2019; Duan et al., 2020; Irfan et al., 2020).

To ensure our study remained commercially relevant, contrasting applications of commercial watercress fertilizer were made in a commercial farm. Despite these treatments also altering N and K application, P remained the limiting macronutrient in P− treatment, in line with what has been reported previously (Crisp, 1970; CaBA CSRG, 2021). This was shown through (a) N concentration remaining constant within the water supply of both treatment beds at harvest point; (b) K concentration of plant tissue being unaffected by treatment but P concentration of plant tissue was lower in P− treatments; and (c) changes to biochemistry typical of P-deficiency were observed in plants grown in P− alongside gene expression changes observed in other species grown in P-deficient conditions.

### 4.2 RNA-seq reveals that cell membrane remodeling dominates genetic basis of PUE in watercress

Mobilizing the resources to ensure continued growth with lower P inputs appears to result from remobilization and scavenging strategies, such as by substituting phospholipids in the cell membrane with other lipids (Yu et al., 2002). This strategy is evident in the DEGs of the P− roots and subsequent GO enrichment analysis, which showed pathways with the highest representation were for sulfolipid and galactolipid biosynthesis (e.g., *SQD1/2*, *DGDG1/2, MGDG3, PLPZETA2*, and *UGP3*). Previous RNA-seq and microarray studies in *A. thaliana* identified genes related to galactolipid biosynthesis as the largest group within a core subset of P-starvation response (PSR) genes, and here we have shown that these genes were most strongly induced in P− watercress (Lan et al., 2015). These genes function to free P from phospholipids, which accounts for 20% of plant P content (Veneklaas et al., 2012). *SQD1/2, DGDG1/2, MGDG3, PLPZETA2*, and *PECP1* were confirmed here to be primary functional genomic targets for developing more P-use efficient watercress (Hibbert and Taylor, 2022).

Other gene homologs important for watercress PUE include those for P transport, such as high-affinity P transporters *PHT1;4* and *PHT1;8*, which were both upregulated in P− roots. The high affinity carnitine uptake transporter *OCT1* was not previously predicted to be important for PUE in watercress; however, it was found as inducible here. This P-inducibility has been shown in other studies and *OCT1* is known to regulate lateral root development (Lelandais-Brière et al., 2007; Lan et al., 2015). One study also found its expression to be affected by *PHO2*, which regulates translocation of P from shoots to roots (Bari et al., 2006). *SPX1* and *SPX3*, transcription factors with multi-functional roles in the PSR, were both upregulated in P-starved roots. SPX-domain-containing genes are part of a core subset of PSR genes in *A. thaliana* and were predicted to be candidates for PUE in watercress, which is confirmed in this study (Lan et al., 2015; Hibbert and Taylor, 2022). Both are upregulated in *A. thaliana* under P starvation and play positive roles in adaptation to P−, with *SPX3* exerting negative feedback regulation of *SPX1* (Duan et al., 2008).

Suberization of roots may also have contributed to the increase in root biomass. Suberization is the deposition of suberin polymers within the root exodermis and endodermis to form hydrophobic barriers (Baxter et al., 2009). GO analysis found that DEGs involved in suberin biosynthesis contributed to a 57-fold enrichment of this pathway in watercress. Suberin has been shown to play a pivotal role in drought, waterlogging, and nutrient homeostasis by providing an apoplastic barrier within the root (Barberon et al., 2016). Studies on effects of P-starvation have found no increase in suberization in P-deficient barley (*Hordeum vulgare*) and lower suberization in *A. thaliana*, in contrast to the suggestion of enhanced suberin biosynthesis in this study, suggesting possible fundamental differences in mechanisms between terrestrial and aquatic plants (Andersen et al., 2018; Grünhofer et al., 2021). Müller et al. (2021) showed varying levels of upregulation of numerous PSR genes following whole plant submergence, which may suggest a link between P starvation and watercress submergence responses. Purple acid phosphatases (PAPs) that are secreted outside roots to mobilize P from bound sources are not relevant to watercress cultivation systems where this free P would rapidly wash away. However, intracellular phosphatases are of interest for PUE in aquatic crops as these remobilize P within the plant. Our study identified upregulation of *PAP17* (AT3G17790) with dual roles in P mobilization and ROS metabolism (del Pozo et al., 1999). Additionally, phosphatases not previously regarded as central for PUE were also upregulated in P− watercress. This includes *DSP2* (AT2G32960), which encodes a tyrosine-specific phosphatase and whose expression was increased 3.2-fold in one study with P-starved *A. thaliana* (Müller et al., 2007). The gene for the inorganic pyrophosphatase PPA4 (AT3G53620), which releases P from pyrophosphate, was also upregulated in watercress here (Navarro-De la Sancha et al., 2007). These results suggest an increased importance for investigating alternative phosphatases that have previously been overlooked in PUE studies. Key to all these changes is differential gene expression of numerous transcription factors associated with the PSR such as *SPX1, SPX3, WRKY75*, and *HHO2* in watercress roots (Puga et al., 2014; Zhou et al., 2015; Wang et al., 2020).

### 4.3 Antioxidant capacity is increased without loss to glucosinolate concentration in P deficiency

AO capacity and GSL concentration are of critical importance as nutritional traits, linked to beneficial impacts on human health and central to the watercress nutritional profile, of relevance to the consumer. AO capacity was increased in P− plants, and there were no changes to the total concentration of GSLs. Increased AO capacity could be linked to the impairment of photosynthetic rate (via impairing electron transfer) in P deprivation, leading to ROS generation, which is countered through increased synthesis of AOs and activity of AO enzymes, such as peroxidase (Meng et al., 2021). This is a widely reported response to P−, with evidence in bean (*Phaseolus vulgaris*), tomato (*Solanum lycopersicum*), rice (*Oryza sativa*), and maize (*Zea mays*) (Juszczuk et al., 2001; Zhang et al., 2014; Muneer and Jeong, 2015; Veronica et al., 2017). The studies in bean and maize also found P− decreased leaf area, as in watercress. Leaf biomass was also higher in phosphate-treated garden sage (*Salvia officinalis*) in agreement with our results, but this was associated with increased leaf phenolic (AOs) concentration (Nell et al., 2009). However, the P+ concentration used by Nell et al. (136 mg L^−1^ PO_4_) far exceeded those used in our field conditions (P+ ~0.06 mg L^−1^). The increase in AO capacity of P− watercress is supported by transcriptomic changes in our study: *VTC4*, involved in ascorbate biosynthesis, was upregulated in P− shoot tissue (Conklin et al., 2006). Interestingly, catalytic activity by VTC4 releases phosphate, so this protein may serve a dual function for PUE by freeing bound P and providing AO scavenging (Torabinejad et al., 2009).

No previous studies exist on the effects of fertilizer application on watercress GSL concentration; however, there is literature for other species. Contrary to our results, GSL concentration of Arabidopsis increased in P− conditions; however, the P− conditions used in that trial (3 µM = 0.285 mg L^−1^ PO_4_) exceed mean P concentration observed in the irrigation supply here, even in P+ conditions in our trial, so these effects may not be relevant at very low P concentrations (Pant et al., 2015). In rocket (*Eruca sativa*), increasing P elevated total GSL concentration (Chun et al., 2017). However, once again, even the lowest P concentration (0.5 mM = 45.49 mg L^−1^ PO_4_) far exceeded those used in this trial. Additionally, GSLs quantified in this study were methionine-derived, whereas gluconasturtiin (comprising >90% of GSL in watercress) is phenylalanine-derived (Underbill, 1965; Voutsina et al., 2016). Considering methionine is a sulfur-containing amino acid and phenylalanine is not, it is possible that GSL concentration not affected in watercress because there is no trade-off with sulfur allocation between GSL biosynthesis and low P-induced cell membrane remodeling (as sulfolipids). These GSL studies also raise the question of how biologically relevant P-deficient conditions are to field conditions. Although the chalk streams supplying watercress farms naturally contain unusually low concentration of phosphate (<0.04 mg L^−1^), P available in solution within soils has been reported to be approximately 0.05 mg L^−1^, but this varies considerably with soil type (Havlin et al., 2005). In several Brassica species (*B. campestris* and *B. juncea*) given either 0.1 mM or 0.5 mM P, a similar result was found to watercress in our study: Trejo-Téllez et al. (2019) found that there were no differences in the concentration of alkenyl-GSLs (the primary GSL group in the plants) and several indole-GSLs between different P supplies in either species tested. However, responses of GSLs to treatment varied between species. This demonstrates the variability of GSL responses to P.

Growers are also interested in developing a sweeter watercress crop to appeal to consumers who find its peppery taste polarizing (Dr. H. Smith, Vitacress, 2020, personal communication). Therefore, it is interesting that sugar and starch concentration were also increased in P− shoots. Increases in starch and sugars is a well-reported response to low P (Rychter and Randall, 1994; Ciereszko and Barbachowska, 2000; Müller et al., 2007; Hammond and White, 2011; Li et al., 2021). Sugar is critical for signaling cascades involved in the P-starvation response and many P-responsive genes are sugar-inducible such as *PHT1;1* (Karthikeyan et al., 2007; Rouached et al., 2010). Starch accumulation is attributed to an increase in Calvin cycle intermediates (due to decreased exchange of triose-phosphate with cytosolic P) from which P can be liberated (MacNeill et al., 2017). It may also be the case in this study that sugar and starch concentrations were higher as they were more concentrated in dwarfed shoots. RNA-seq results also support the role of sugars in P starvation responses. *CTIMC* (AT3G55440), involved in gluconeogenesis, had a log fold-change of 0.45 (*p* < 0.0001) in P− shoots compared to P+ shoots in our study (Dumont et al., 2016). An increase in the CTIMC protein triosephosphate isomerase has been reported to increase in P− maize roots (Li et al., 2007a). SEX4 dephosphorylates starch granules to regulate starch accumulation, and its gene was also upregulated here (Kötting et al., 2009). In P− watercress roots, *GWD1* (AT1G10760), which regulates the addition of P to starch, was downregulated (Yu et al., 2001).

It is promising that there was no compromise to the nutritional quality, but the peppery flavor of glucosinolates could be softened by increased sugars. Taste trials are needed to quantify the effects of enhanced sugars on consumer taste preferences. Importantly, increased AO capacity and the maintenance of GSL concentration for commercial crops grown with less P input provide a win–win situation for watercress, with maintained or enhanced nutritional characteristics and an improved environmental footprint. Additional trials are also required to see if these effects are stable across different environments and growing seasons.

### 4.4 Variation in PUE enables breeding for PUE in watercress

To further explore the effects of P deprivation on watercress morphology and biochemistry, an additional aim of this study was to use variation in these responses to P to inform selections for future breeding. Stem length, number of leaves, and individual leaf area all varied significantly between lines and ranking of lines by trait means, and assessing percentage change in response to low P is valuable for making initial line selections for future breeding. This showed that lines 60 and 102 were valuable for quantification of P and K concentration and differential gene expression analysis by RNA-seq. Line 60 was P-responsive (e.g., 114% increase in mean root fresh weight in P−) and had a relatively high AO capacity, whereas line 102 ranked the highest for several yield traits while exhibiting low responsiveness to P treatment. Overall, PUE was the highest in line 60 followed by 102, as reflected by the highest P concentration in dried shoot tissue under both conditions compared to the commercial control. This provides further evidence to progress these lines in breeding for PUE in watercress.

The large increase in root biomass for line 60 in P− suggests that the PUE strategy for this line focuses on altered root morphology, or this could be a by-product of effective P remobilization strategies, whereas the relatively P-unresponsive yield seen in 102 suggests enhanced uptake (e.g., higher activity of P transporters) and more effective utilization internally. The development genes (*UGE4, MYO6*, and *PSP*) upregulated in P− in line 60 indicate morphology changes. However, of these, only *MYO6* upregulation was unique to line 60 (*PSP* and *UGE4* were also upregulated in 102, with respect to WXVITA). Line 60 has also been shown to have consistently high GSL concentration and leaf area in previous trials in the UK and US (Qian et al., 2023). In addition, DEGs identified in both lines were involved in phosphate ion homeostasis (*CAX1* and *UBC24*, aka *PHO2*). The downregulation of *PHO2* in lines 60 and 102 (with respect to WXVITA) in P− is especially interesting as *PHO2* downregulation results in reduced degradation of PHO1, leading to increased P loading to the shoots (Liu et al., 2012). This could partly explain the higher P concentration and yield of these selected lines compared to WXVITA. These genes provide further targets for breeding watercress with improved PUE. The identification of *PHO2* in these selected lines with improved PUE also supports our previous review that listed *PHO2* as a candidate gene for PUE breeding in watercress (Hibbert and Taylor, 2022).

## 5 Conclusion

**Figure 9:**
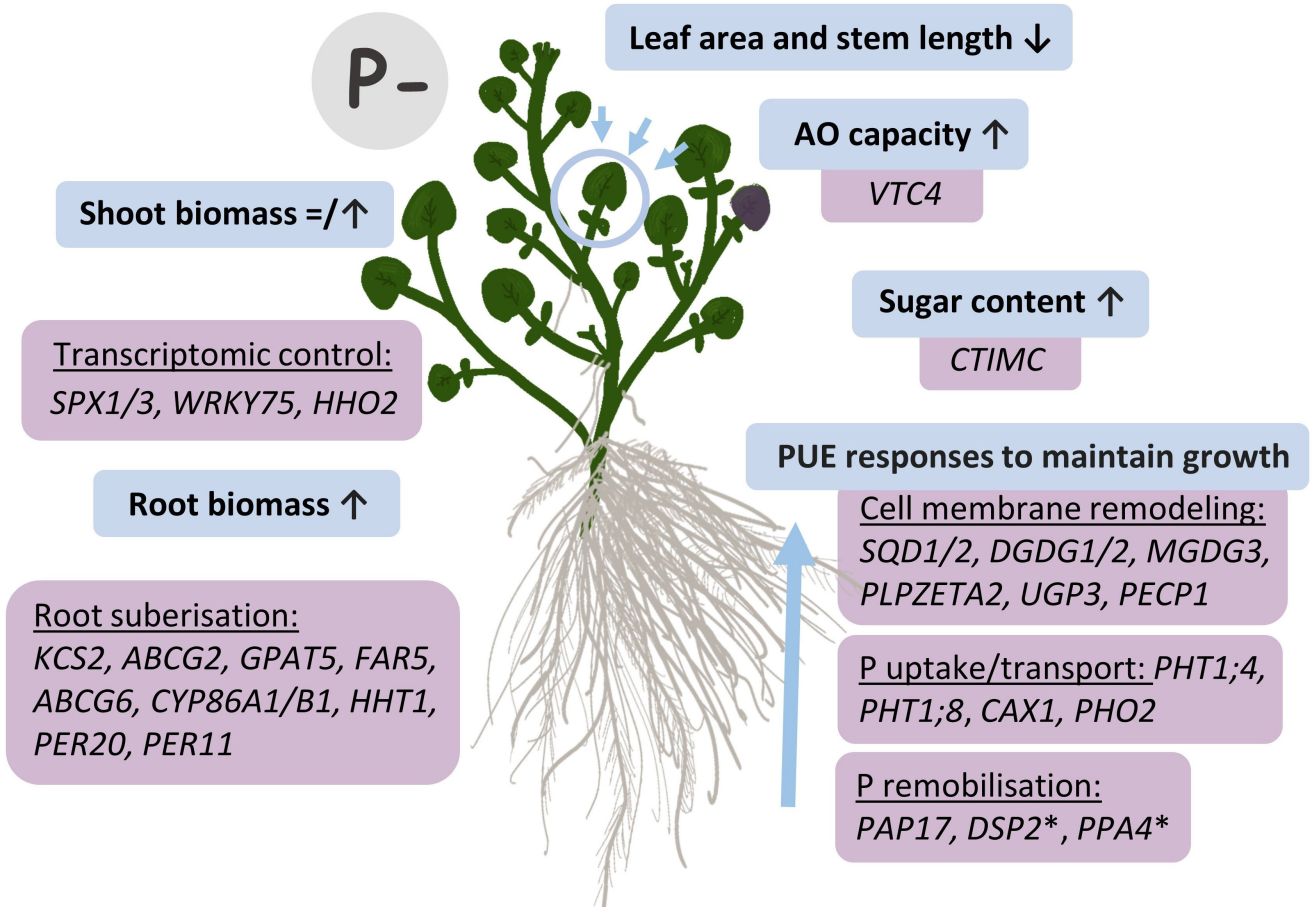
Responses to P deficiency (P−) in watercress and genetic targets identified by RNA-seq. Blue boxes illustrate morphological and biochemical changes observed in P− conditions, with respective genes associated with these changes and identified through RNA-seq results in purple boxes. Up (↑) arrows denote an increase, whereas ↓ arrows denote a decrease. The = icon illustrates the absence of an effect of P− on shoot fresh biomass whereas shoot dry biomass increased. *Other unexplored phosphatases identified here but not previously recognized as PUE genes in *A. thaliana* that could provide PUE roles.

We provide the first report on the effects of contrasting phosphate-based fertilizer treatments on the morphology, biochemistry, and transcriptome of watercress, alongside the identification of a suite of genes important for PUE in this aquatic species. Taken together, this information will underpin future watercress breeding (**Figure 9**). Watercress plants sustained shoot yield without additional fertilizer treatment, partially through enhanced root biomass, and had shorter stems and more densely packed leaf area. Strategies (and associated genes) for improved low P tolerance in watercress focused on cell membrane remodeling (e.g., *SQD1/2, DGDG1/2, MGDG3, PLPZETA2, UGP3*, and *PECP1*), also regarded as a core gene set for the PSR in *A. thaliana* (Lan et al., 2015). Homologs of other known PUE genes such as P transporters (e.g., *PHT1* and *PHO2*), transcription factors (e.g., *SPX1/3, WRK75*, and *HHO2*), and phosphatases (e.g., *PAP17*) were also identified as important for watercress PUE. Additional genes not previously regarded as important for their PUE function included numerous genes for root suberization, suggesting a higher emphasis on this strategy for PUE in aquatic crops like watercress. Overall, this study identified new germplasm and genetic targets to assist with advancing PUE in watercress breeding.

## Data availability statement

The data presented in this study are deposited in NCBI's Sequence Read Archive (SRA), BioProject number PRJNA941001.

## Author contributions

LH: Data curation, Formal Analysis, Investigation, Methodology, Software, Visualization, Writing – original draft, Writing – review & editing. YQ: Formal Analysis, Methodology, Software, Writing – review & editing. HS: Investigation, Resources, Writing – review & editing. SM: Investigation, Resources, Writing – review & editing. EK: Formal Analysis, Investigation, Methodology, Writing – review & editing. DK: Funding acquisition, Methodology, Resources, Writing – review & editing. GT: Conceptualization, Funding acquisition, Project administration, Resources, Supervision, Writing – review & editing.
