# Supplementary material for: Making watercress (Nasturtium officinale) cropping sustainable: genomic insights into enhanced phosphorus use efficiency in an aquatic crop
Source: Front Plant Sci. 2023 Nov 7;14:1279823. doi: 10.3389/fpls.2023.1279823 (PMC10662076; doi:10.3389/fpls.2023.1279823)

Supplementary information (SI): Making watercress (*Nasturtium officinale*) cropping sustainable: genomic insights into enhanced phosphorus use efficiency in an aquatic crop

Supplementary 1: Details of commercial fertilizer regime applied in P+ treatment.

Seeds sown in peat (80:20 Baltic white: Baltic black peat). After transplanting, one dose of base dressing (Eco-Cress Base; Humber Palmer) on day 16, then one dose of top dressing (Eco-Cress Plus; Humber Palmer) on day 23 were applied. Due to seed restraints, both plots were planted at ~40 % of the planting density of a commercial bed and thus fertilizer treatments in the P+ treatment were scaled to reflect this. Thus, the application rates were scaled to 200 kg ha-1 of base dressing (13.28 kg ha-1 P2O5) and 100 kg ha-1 of top dressing (5.24 kg ha-1 P).


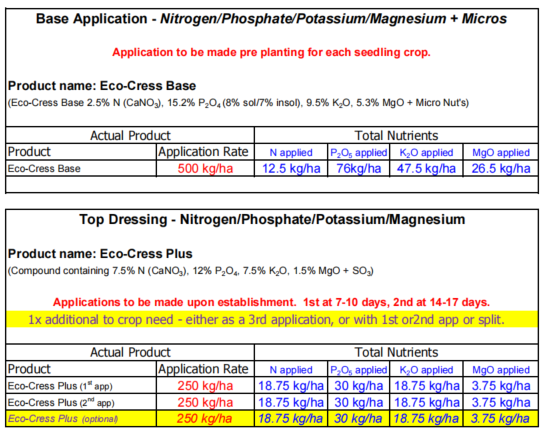


Supplementary 2: Morphology of selected watercress lines (60, 102 and the commercial control, WXVITA) grown under a commercial phosphate-based fertilizer regime (P+) or without additional fertilizer (P-). Images of dissected plants were taken at harvest point (35 days post-transplanting) from plants in block 1.

|  | **Treatment** | |
| --- | --- | --- |
| **Line** | **P-** | **P+** |
| **60** | 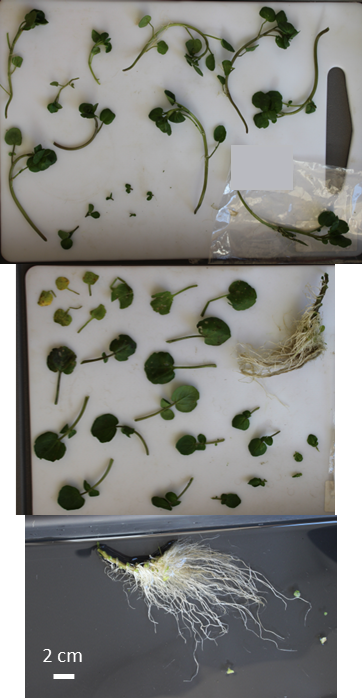 | 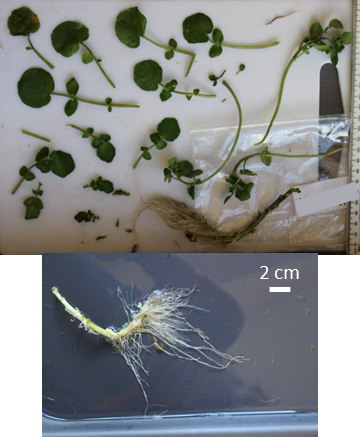 |
| **102** | 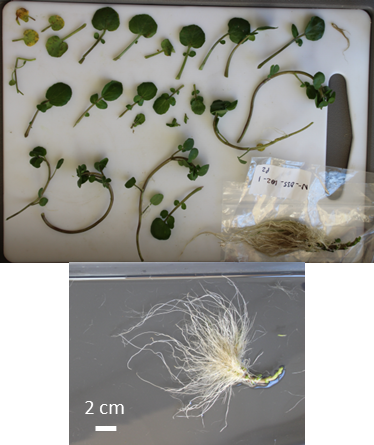 | 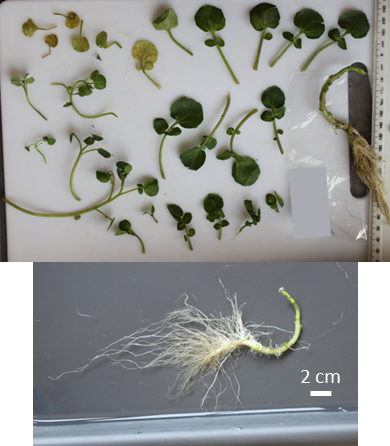 |
| **WXVITA** | 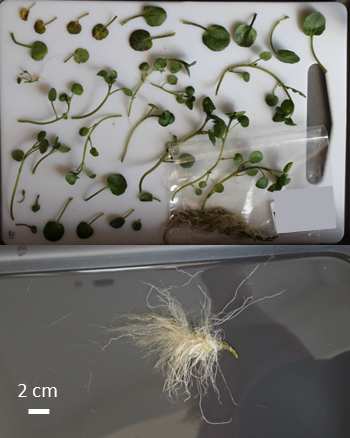 | 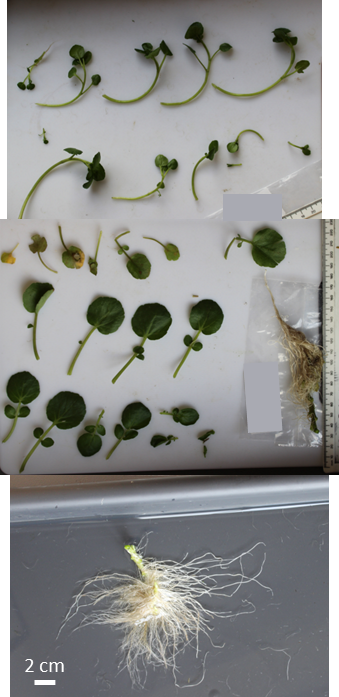 |

Supplementary 3: Genetic variation between lines for additional morphological and biochemical traits


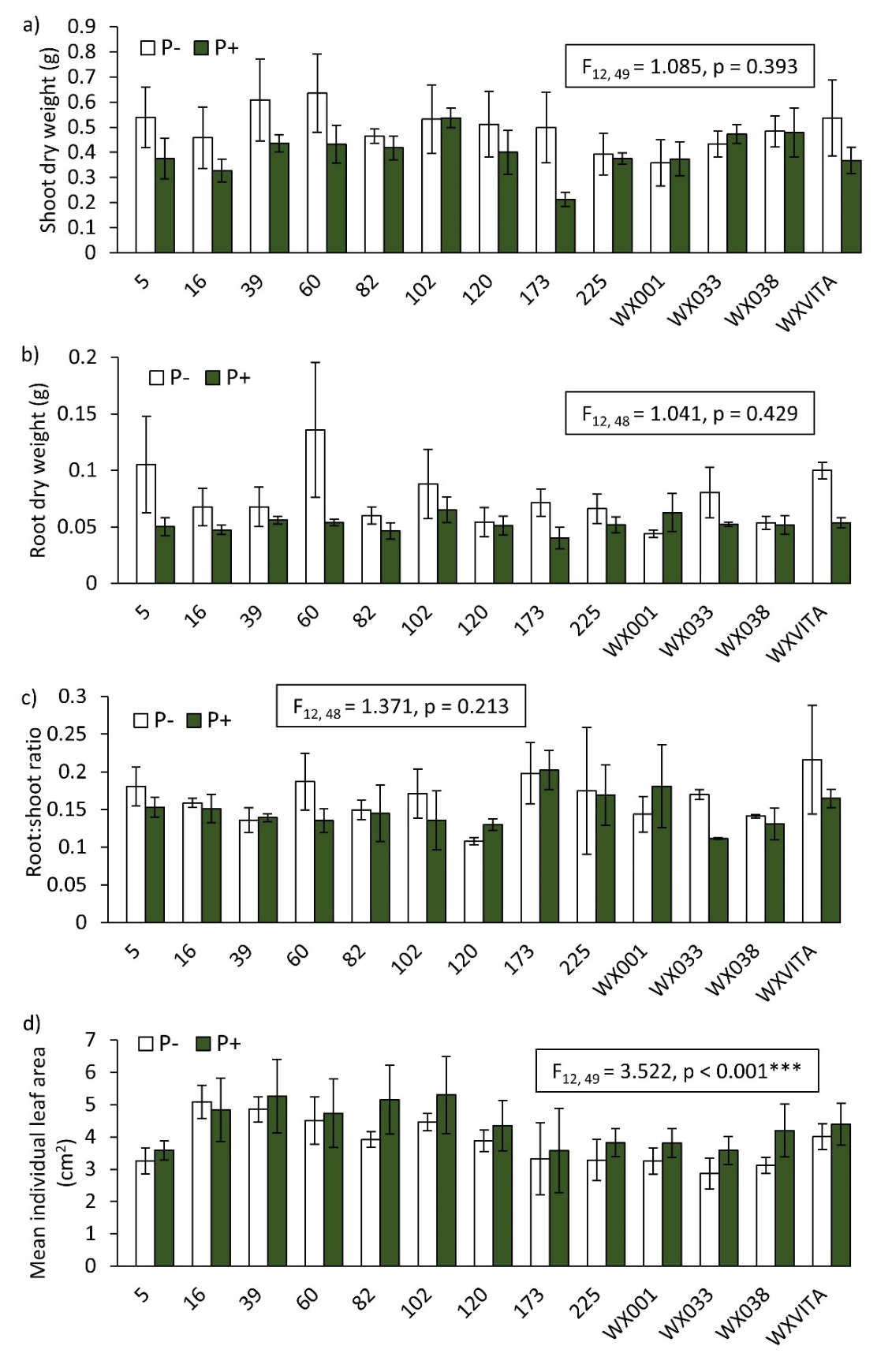
Error bars represent SEM (n = 3). Statistics for the effect of line on each trait (from ANOVA tests on linear models) are given in the text boxes. Line effects are significant at the following levels: *p < 0.05, **p < 0.01, ***p< 0 .001.


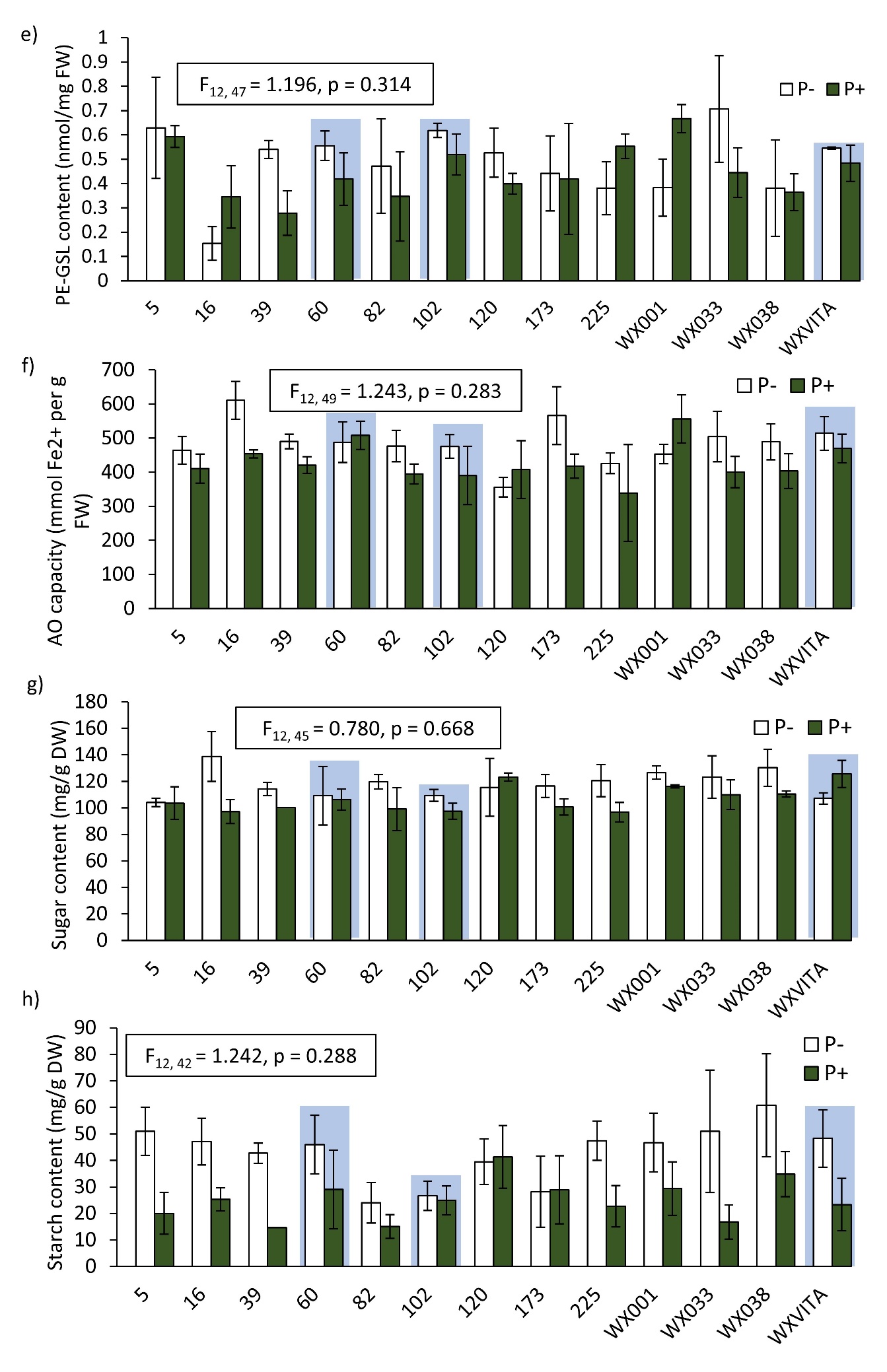


Supplementary 4: top DEGs in P- shoots (compared to P+ shoots). Includes genes identified with both FDR < 0.15 and p value < 0.0001. – see additional file S3

Supplementary 5: full list of annotated DEGs in P- roots (compared to P+ roots) – see additional file S4

Supplementary 6: list of genes differentially expressed between selected lines (60, 102 and commercial control WXVITA) in root (R) and shoot (S) tissue, from watercress grown with phosphate-based fertilizer (P+) or without (P-). Where watercress transcripts matched an annotation from Uniprot, this is given in column H.

Supplementary 7: Number of genes that are a) upregulated and b) downregulated in roots of selected lines (102 and 60) grown with phosphate-based fertilizer (P+) or without (P-). Differentially expressed genes are respective to expression in WXVITA. Numbers in paratheses indicate the percentage of DEGs from each group falling into each category. Venn diagrams made using VENNY.


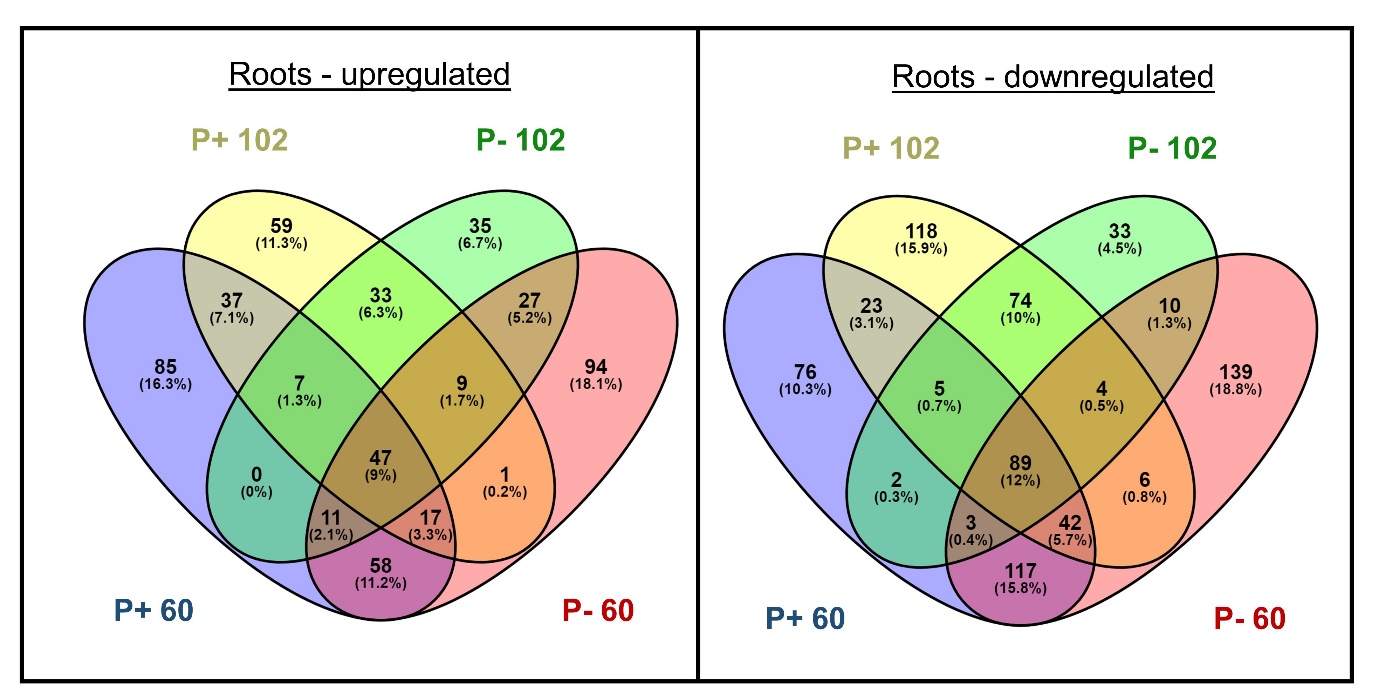


Supplementary 8: Top enriched GO biological processes of DEGs from different line comparisons.

Point size reflects the number of genes with the corresponding GO term and line color represents –log10(FDR). Fold enrichment is defined as the percentage of genes belonging to a pathway, divided by the corresponding percentage in the background.

1.
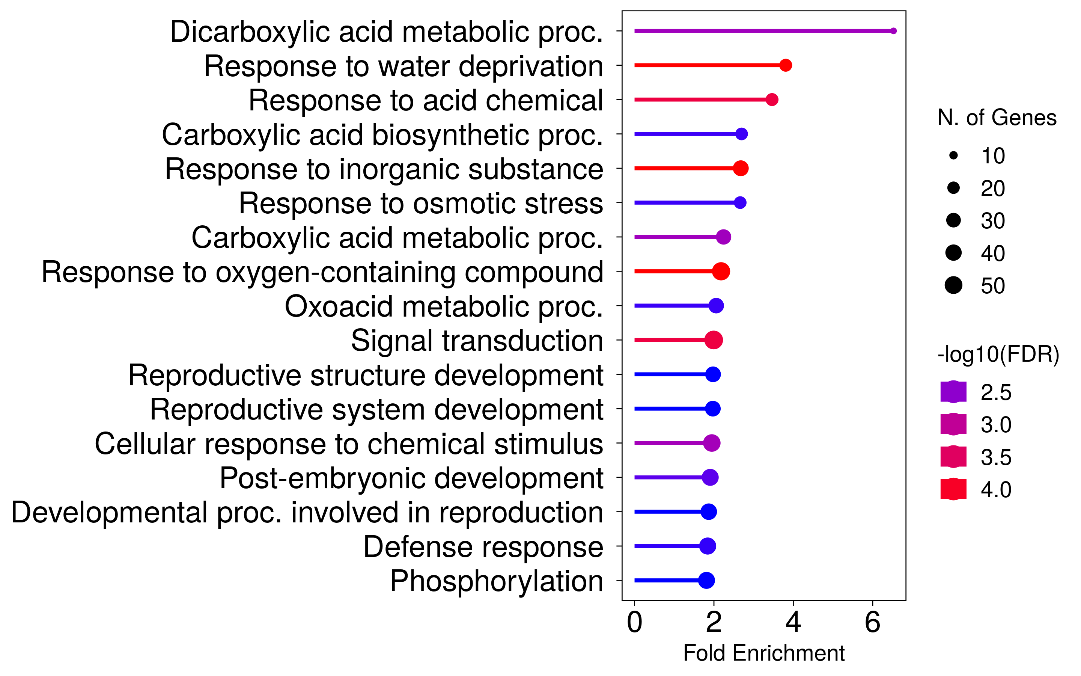
P- roots: 60 vs WXVITA


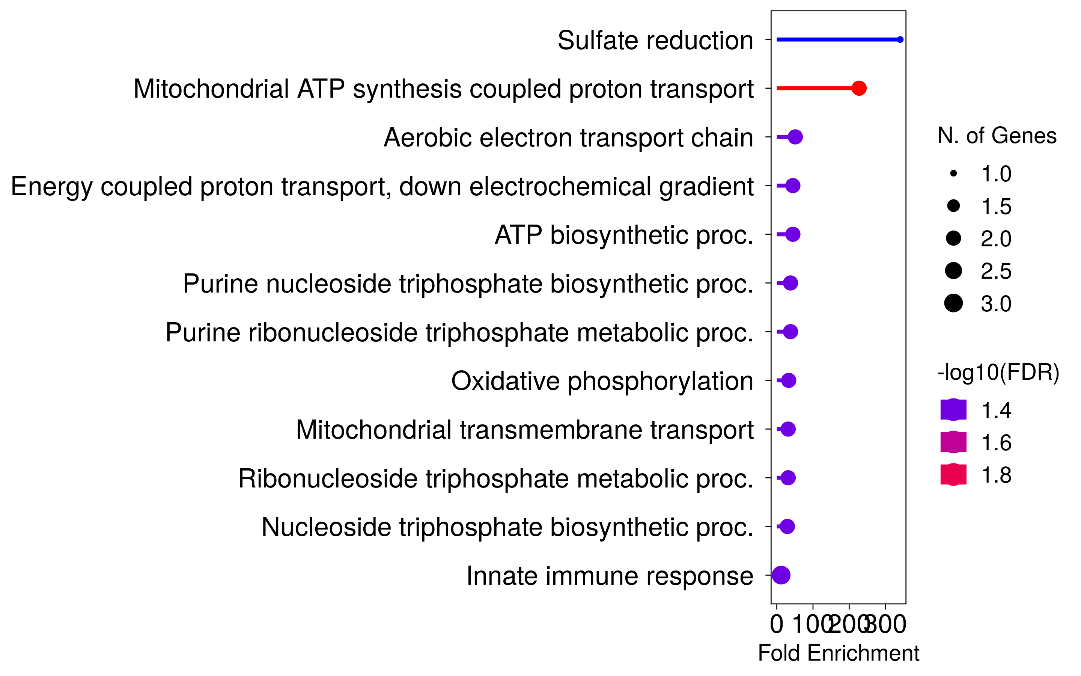


1. P- roots: 102 vs WXVITA
2.
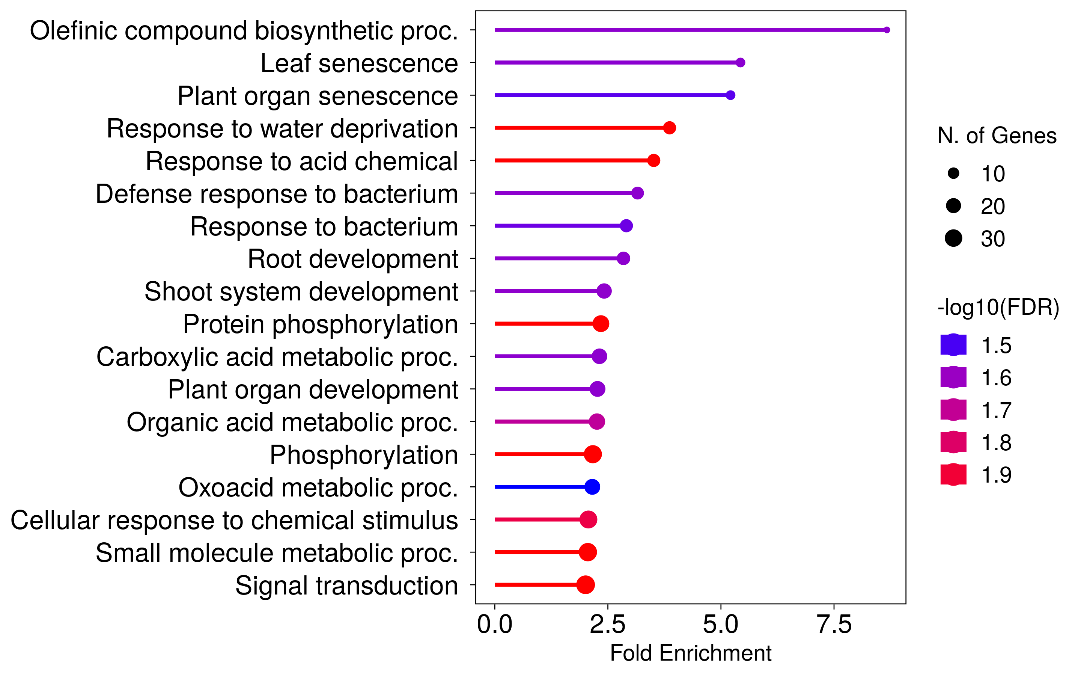
P- roots: genes DE in both 60 and 102 vs WXVITA


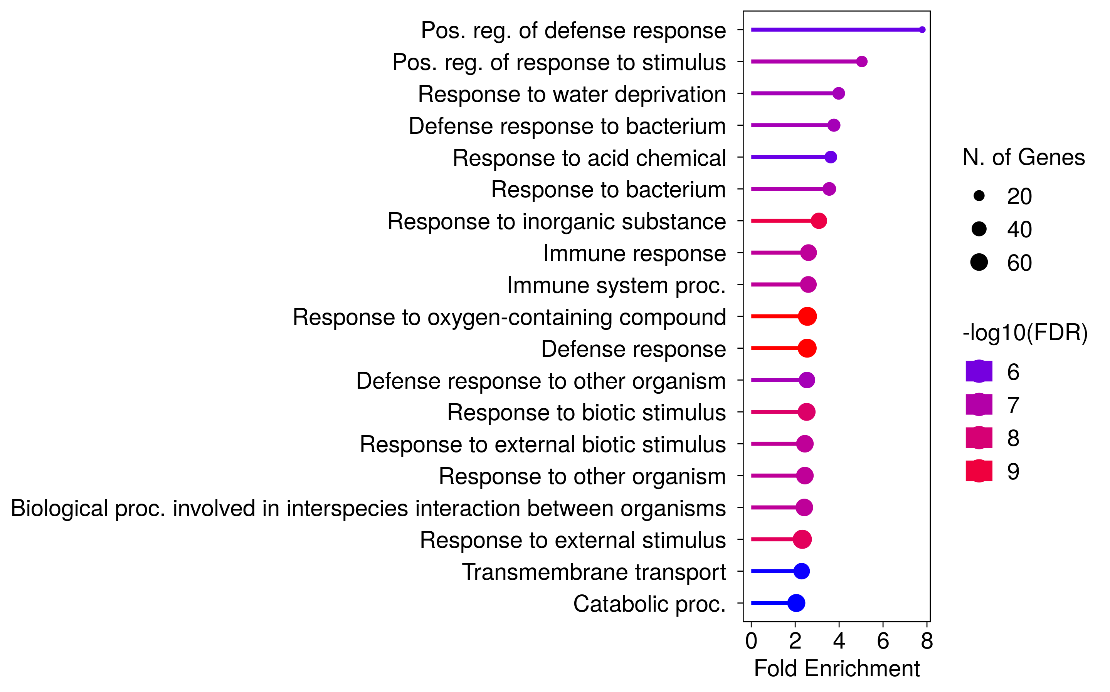


1. P- shoots: 60 vs WXVITA


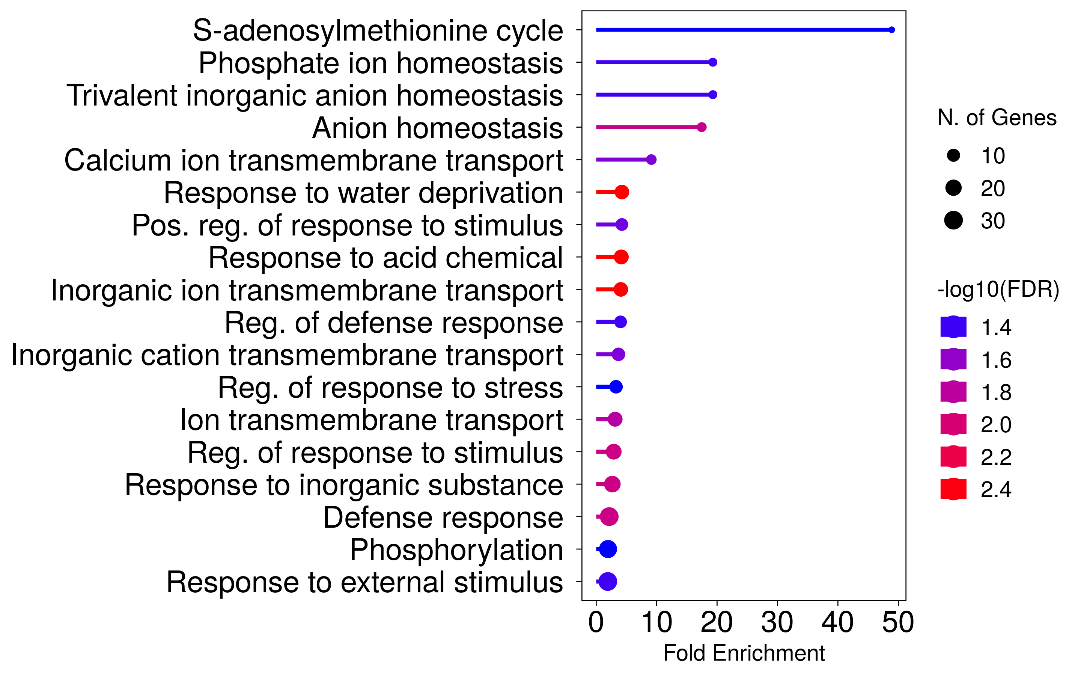


1. P- shoots: 102 vs WXVITA
2.
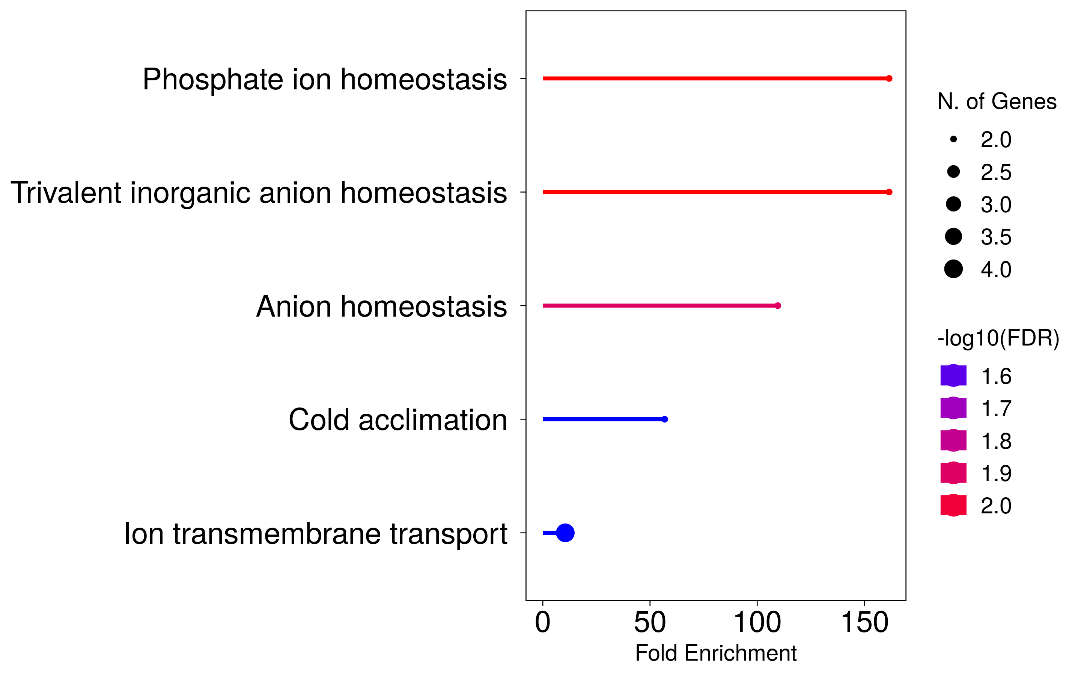
P- shoots: genes DE in both 60 and 102 vs WXVITA

Supplementary 9: Contribution of basal and adventitious roots to total root biomass.

Dissected watercress stem and root system, showing extensive adventitious root formation at day 35 (b) compared to day 15 (a). Images were taken from the same plot (P-, block 1, line 60). The basal root (circled) only composed ~37 % of root biomass at day 15 (c). The schematic (d) illustrates the partitioning of the root system within a commercial growing system, with a small basal root (yellow) in the gravel substrate, and prolific adventitious root growth (deriving from the stem/leaf nodes) into the substrate (up to approx. 5 cm deep) and water column.


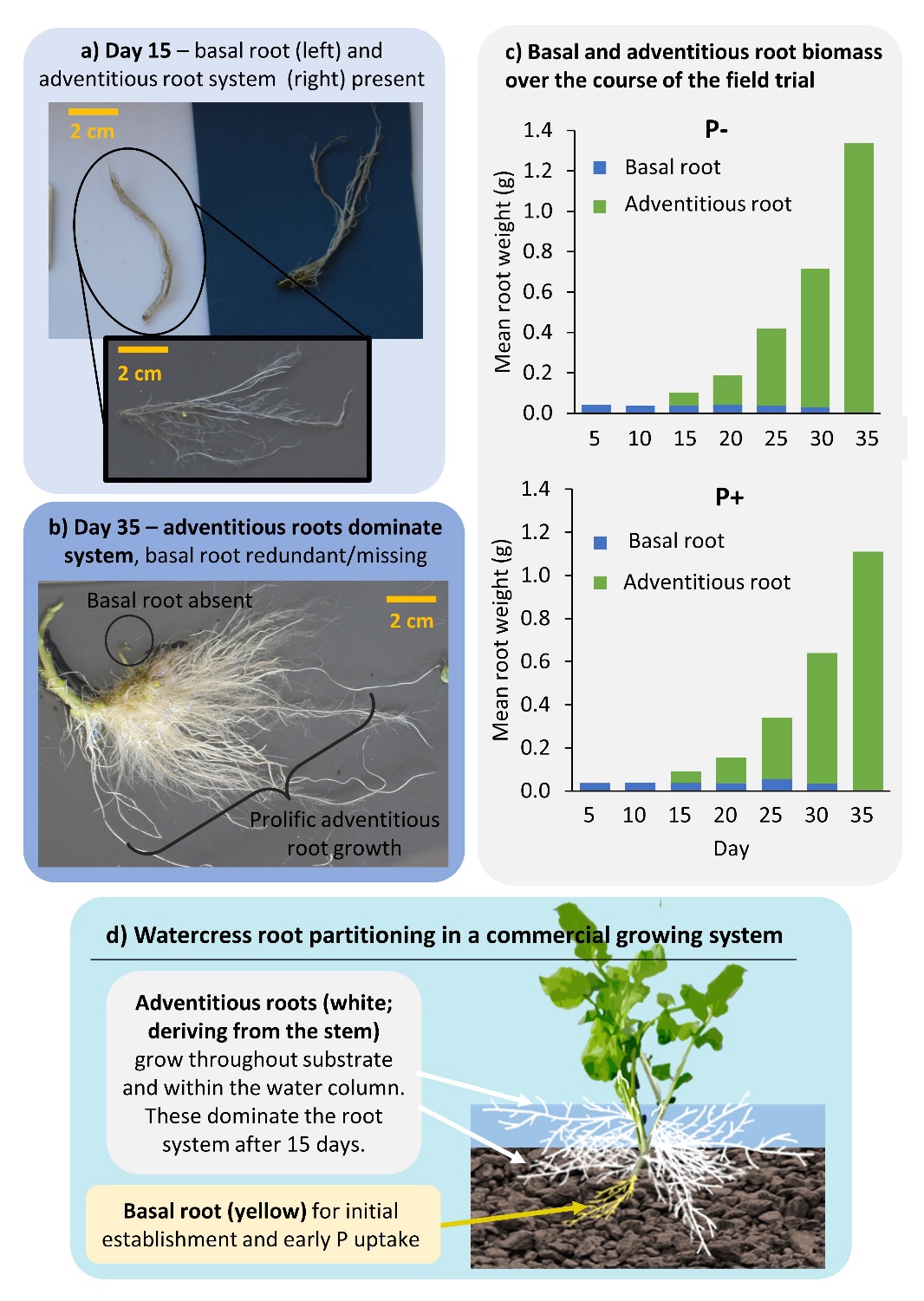

Supplement: Supplementary file 1 [file DataSheet_1.zip › Supplementary materials/Supplementary 1-3 and 7-9.docx]
